# Automatic detection of gastrointestinal system abnormalities using deep learning-based segmentation and classification methods

**DOI:** 10.1007/s13755-025-00354-6

**Published:** 2025-05-21

**Authors:** Abdullah Şener, Burhan Ergen

**Affiliations:** 1 Management Information Systems, Faculty of Economics and Administrative Sciences, Fırat University, Elazığ, 23100 Turkey; 2 Computer Engineering, Faculty of Engineering, Fırat University, Elazığ, 23100 Turkey

**Keywords:** Gastrointestinal system abnormalities, GISegNet, Deep learning-based segmentation, Automated classification and diagnosis, Endoscopic ımage analysis

## Abstract

Early diagnosis and precise treatment of gastrointestinal (GI) diseases are crucial for reducing mortality and improving quality of life. In this context, the detection and classification of abnormalities in endoscopic images is an important support for specialists during the diagnostic process. In this study, an innovative deep learning approach for the segmentation and classification of pathological regions in the GI system is presented. In the first phase of the study, a novel segmentation network called GISegNet was developed. GISegNet is a deep learning-based architecture tailored for accurate detection of anomalies in the GI system. Experiments conducted on the Kvasir dataset showed that GISegNet achieved excellent results on performance metrics such as Jaccard and Dice coefficients and outperformed other segmentation models with a higher accuracy rate (93.16%). In the second phase, a hybrid deep learning method was proposed for classifying anomalies in the GI system. The features extracted from the transformer-based models were fused and optimized using the Minimum Redundancy Maximum Relevance (mRMR) algorithm. The classification process was performed using Support Vector Machines (SVM). As a result of feature fusion and selection, the second model, which combined features from DeiT and ViT models, achieved the best performance with an accuracy rate of 95.2%. By selecting a subset of 300 features optimized by the mRMR algorithm, the accuracy (95.3%) was maintained while optimizing the computational cost. These results show that the proposed deep learning approaches can serve as reliable tools for the detection and classification of diseases of the GI system.

## Introduction

Gastrointestinal diseases such as ulcers, polyps, Crohn’s disease, cancer and bleeding are common diseases of the human digestive system and pose a significant health threat worldwide [1]. These diseases can severely affect an individual’s quality of life and, in advanced stages, can lead to life-threatening complications. Globally, oesophageal, gastric, and colorectal cancers rank among the most prevalent cancer types and are notable for their high mortality rates [2]. This underlines the crucial importance of early diagnosis and effective treatment of these diseases. Endoscopic methods used in the diagnosis and treatment of digestive tract diseases provide a highly effective medical imaging technique for the detection of abnormalities. Endoscopy is a minimally invasive procedure in which internal organs and tissues are directly visualized using a flexible, thin and elongated tube called an endoscope [3]. The endoscope, which is equipped with a light source and a camera, transmits detailed images of the internal organs to a monitor. This procedure is performed through natural or minimally invasive entry points such as the mouth, nose or small incisions in the skin [4]. Endoscopy not only facilitates the detection of abnormalities, but also enables certain therapeutic procedures to be performed. Operations such as the removal of polyps, the taking of biopsies or the control of bleeding can be performed during an endoscopic examination. This reduces the need for surgery, increases patient comfort and shortens recovery time. This essential instrument of contemporary medicine has established itself as a standard approach for diagnosing gastrointestinal disorders, significantly contributing to enhanced patient survival rates and quality of life [5].

Timely and accurate diagnosis of abnormalities in the human gastrointestinal system is crucial for reducing the mortality rate. Endoscopic procedures are vital for identifying such abnormalities, as they aid not only in diagnosing the disease but also in evaluating its severity and determining suitable treatment strategies. Conducted by gastroenterology specialists, these examinations deliver precise and comprehensive information about the patient’s gastrointestinal condition [6]. They contribute significantly to clinical decision-making, prevent disease progression and improve patient health outcomes. However, image and video analysis performed by gastroenterologists in the context of the GI system may vary from one specialist to another due to differences in clinical experience and individual approaches. This variability in the interpretation of endoscopic images and diagnostic videos can occasionally lead to misdiagnosis. To minimize such diagnostic errors and standardize diagnostic processes, automatic classification and segmentation methods for detecting GI abnormalities from endoscopic images are widely used.

This study aims to develop an efficient deep learning-based segmentation and classification system to improve the automated detection of gastrointestinal diseases, enhancing diagnostic accuracy and reducing workload for specialists. The proposed system is designed to assist specialists by automating the segmentation and classification process, reducing the burden of manual diagnosis and minimizing human errors. In the initial phase, a specialized segmentation network, termed GISegNet, was designed to accurately segment complex pathological regions. GISegNet employs a deep learning-based framework tailored for the precise identification of pathological areas in the GI system. The architecture is composed of two key components: an encoder, which extracts detailed features from input images to generate deep feature representations, and a decoder, which refines these features to produce accurate segmentation outputs.

A key innovation of GISegNet is the introduction of the Encoder-Decoder Redundancy (EDR) block. This block utilizes asymmetrical convolutional layers to extract detailed local features while simultaneously enriching contextual information, enhancing the model’s capacity to capture feature relationships and deliver more precise segmentation results. Moreover, the model incorporates the Efficient Hybrid Attentional Atrous Convolution (EHAAC) mechanism, which integrates spatial and channel-based attention techniques to highlight critical features and filter out irrelevant information. The use of skip connections between the encoder and decoder, facilitated through the EDR block, further enhances the model’s segmentation accuracy and consistency. Additionally, the decoder employs transpose convolution layers to upsample low-resolution feature maps from the encoder into high-resolution outputs, thereby improving the level of detail in the segmentation maps.

In the second phase of the study, a hybrid deep learning approach was proposed for the classification of abnormalities in the GI system. During this process, preprocessing steps were applied to the dataset to improve data quality for training transformer-based models. Features generated in the fully connected (FC) layers of the transformer models were combined with information from different models to create a more comprehensive and representative feature set. This expanded feature set was then optimized using the Minimum Redundancy Maximum Relevance (mRMR) algorithm, ensuring the selection of only meaningful and independent features. For classification, Support Vector Machines (SVM) were employed to enhance the model’s accuracy and generalization performance. This approach aimed to reduce computational costs while increasing classification performance by using fewer features.

The main contributions of this research are summarized as follows:

- Development of a novel deep learning segmentation network, GISegNet, for detecting abnormalities in the human GI system.
- The development and integration of the Encoder-Decoder Redundancy (EDR) block and the Efficient Hybrid Attentional Atrous Convolution (EHAAC) mechanism to enhance segmentation accuracy and performance.
- Proposal of a hybrid approach for classification using transformer models, enabling synergistic combination of features derived from multiple models.
- Optimization of feature selection processes with the Minimum Redundancy Maximum Relevance (mRMR) algorithm.
- Implementation of SVM for classification, enhancing accuracy, generalization, and computational efficiency.
- Demonstration of high accuracy and consistency in both segmentation and classification processes, significantly contributing to the analysis of GI system pathologies.

The structure of the paper is organized as follows: Sect. “Literature review” reviews the literature in the field. Sect. “Materials and methods” presents the dataset and methodologies employed in this study. Sect. “Experimental results and discussion” discusses the experimental results and provides a detailed analysis. Sect. “Conclusion and future work” summarizes the key findings and conclusions, and outlines potential directions for future research.

## Literature review

Deep learning (DL) methods have been of great interest in medical image analysis, especially in gastroenterology. One of the main goals of these studies is to improve the diagnosis of gastrointestinal (GI) diseases based on endoscopic images. Manual assessment of these images is time-consuming and needs extensive expertise, which is not straightforward in clinical practice. To overcome these limitations, researchers have developed effective DL architectures, especially CNNs, to enable automatic diagnosis. Through convolutional operations that extract and examine image features, such approaches not only enhance the precision of diagnosis but also reduce the workload of the experts, consequently resulting in more efficient treatment recommendations. Along with these developments, the reviews of the literature highlight the immense success of deep learning-based classification and segmentation in rapid and precise detection of GI disorders. The methods were shown to be trustworthy in assisting gastroenterologists with reducing the risk of misdiagnosis and streamlining clinical decision-making processes. Demirbaş et al. [7] introduced a novel architecture called Spatial-Attention ConvMixer (SAC) to improve the automatic classification of gastrointestinal (GI) diseases. When tested on the Kvasir dataset, SAC showed superior performance in disease detection by employing a spatial attention mechanism and achieving high accuracy rates. Pal et al. [8] proposed a hybrid model called U-MaskNet for classification and segmentation of different GI cancers. U-MaskNet integrates the architectures of U-Net and Mask R-CNN to enable both pixel-level classification and instance segmentation. Experiments performed on the Kvasir dataset showed that U-MaskNet outperformed other models such as DeepLabv3 + and FCN. Zhang et al. [9] proposed an innovative approach to automate the segmentation of gastrointestinal (GI) regions in magnetic resonance imaging (MRI) images. Their model integrates Inception-V4 for classification, a VGG19 encoder with UNet +  + for 2.5D data and Edge UNet for grayscale segmentation. Advanced data pre-processing, including 2.5D processing, improved the accuracy and robustness of the model. This approach provides a comprehensive and efficient solution to the manual and time-consuming segmentation processes in radiotherapy planning. Asif [10] proposed a Fuzzy Minkowski Distance-Based Unified Model for the accurate classification of GI diseases. This model integrates three pre-trained deep learning models—MobileNet, ResNet101 V2, and Xception—enhanced with ResNeXt blocks for feature extraction. The probabilities obtained were combined using the Fuzzy Minkowski Distance method to minimize error rates and improve classification accuracy. Tests on a dataset of 6000 endoscopy images demonstrated the model’s superior accuracy. Maity et al. [11] developed a method using Yolov5 and DeepLabV3 + for the rapid and accurate detection of gastroesophageal reflux disease (GERD) from video endoscopy images. After detecting abnormal regions and performing segmentation, machine learning classifiers improved accuracy rates. Ahamed et al. [12] presented a deep learning-based approach for the automatic classification of GI diseases using the GastroVision dataset. Their method introduced the PD-CNN lightweight feature extractor integrated with the Ensemble Extreme Learning Machine (EELM) classifier enhanced by PCC. The model demonstrated superior performance in metrics such as accuracy, recall, and F1 score. Moreover, it incorporated explainable AI (XAI) techniques, enabling the interpretation of decision-making processes. Khan et al. [13] proposed a model combining deep feature extraction and optimization methods for the automatic detection and classification of artifacts in endoscopic images. Tested on the Kvasir-V2 and CUI Wah datasets, the model employed features extracted from Darknet-53 and Xception networks, optimized using the Binary Dragonfly Algorithm (BDA), and utilized in the ESKNN classifier. Sharma et al. [14] introduced the UMobileNetV2 model for the semantic segmentation of the stomach, small intestine, and colon in MRI scans of GI cancer patients. Combining a MobileNetV2-based encoder and a UNet-based decoder, the model demonstrated strong performance metrics when evaluated on the UW-Madison dataset. Siddiqui et al. [15] proposed a deep learning-based framework for diagnosing and treating GI system diseases. This CNN-based model, working with wireless capsule endoscopy data, integrated feature selection methods to enhance classification accuracy and achieved high performance metrics. Karthikha et al. [16] developed a deep learning-based method called Dilated-U-Net-Seg to address the challenge of accurate polyp segmentation in the early detection of colorectal cancer (CRC). By combining dilated convolutions and feature fusion within an encoder-decoder architecture, their framework achieved a 3.05% improvement in pixel accuracy over existing U-Net architectures. Rajasekar et al. [17] proposed AdaptUNet, a specialized U-Net architecture for accurate polyp detection, incorporating attention mechanisms and wavelet transforms to enhance performance. The model demonstrated superior results across various datasets, improving detection accuracy and reducing manual workload. Singh and Sengar [18] introduced BetterNet, a lightweight and efficient CNN architecture for the early diagnosis of colorectal cancer. Combining residual learning and attention mechanisms, BetterNet achieved high accuracy in polyp segmentation while maintaining computational efficiency. The model outperformed existing methods on datasets like Kvasir-SEG, CVC ClinicDB, and Endoscene and was noted for its suitability for real-time applications. Nguyen et al. [19] proposed ADSNet, a new architecture to address uncertainties in limited regions and improve weak features in polyp segmentation. Featuring a complementary triplet decoder for generating early-stage global maps and a continuous attention module for enhancing high-level feature semantics, ADSNet demonstrated superior performance compared to existing methods and adaptability to other CNN or transformer-based architectures. El-Ghany et al. [20] introduced the Intelligent Learning Rate Controller (ILRC) mechanism for wireless capsule endoscopy (WCE) images to improve the accuracy of detecting GI anomalies, achieving superior results compared to existing methods. Jagarajan and Jayaraman [21] proposed a novel automated diagnostic method supported by a Coati optimization algorithm and hybrid U-Net model, integrating segmentation and classification steps. Following segmentation, classification was performed using Mask RCNN, achieving high accuracy rates and improving diagnostic speed and precision. Siddiqui et al. [15] developed a robust CNN-based framework for diagnosing GI diseases, incorporating feature selection methods to enhance classification rates. The framework was evaluated using various performance metrics, including accuracy, recall, F1 score, mean absolute error, and mean squared error. A summary of the studies in the literature is presented in Table 1.

**Table 1:** Summary of recent studies on deep learning-based detection, classification, and segmentation methods for gastrointestinal (GI) diseases

Authors	Year	Dataset	Decision method
Demirbaş et al. [7]	2024	Kvasir dataset	Spatial-Attention ConvMixer (SAC)
Pal et al. [8]	2024	Kvasir dataset	U-MaskNet
Zhang et al. [9]	2024	Not specified	Inception-V4,VGG19 and UNet + +,
Asif [10]	2024	Kvasir dataset	Fuzzy Minkowski Distance-based Ensemble Model
Maity et al. [11]	2024	Kvasir dataset	Yolov5 and DeepLabV3 +
Ahamed et al. [12]	2024	Kvasir dataset	Ensemble Extreme Learning Machine
Khan et al. [13]	2024	Kvasir dataset and CUI Wah	Darknet-53, Xception, Binary Dragonfly Algorithm (BDA), Ensemble Subspace K-Nearest Neighbors (ESKNN)
Sharma et al. [14]	2024	UW-Madison dataset	UMobileNetV2
Siddiqui et al. [15]	2024	Kvasir dataset	CG-Net:
Karthikha et al. [16]	2024	Kvasir dataset	Dilated-U-Net-Seg
Rajasekar et al. [17]	2024	Kvasir-SEG and Etis-LaribDB	AdaptUNet
Singh and Sengar [18]	2024	Kvasir-SEG, CVC ClinicDB and Endoscene	BetterNet
Nguyen et al. [19]	2024	Kvasir-Seg, CVC-ClinicDB, ETIS, CVC-ColonDB	ADSNet
El-Ghany et al. [20]	2024	Kvasir-Capsule and KVASIR v2	ILRC-Enhanced Deep Learning Framework
Jagarajan and Jayaraman [21]	2024	Kvasir dataset	Coati Optimization and Hybrid U-Net with Mask RCNN Framework

The aforementioned studies have made significant advancements in the utilization of deep learning methods for GI disease diagnosis and segmentation; however, these works also present certain limitations. Many approaches rely on complex architectures that involve high computational costs, thus diminishing their feasibility for real-time clinical applications. In addition, many models exhibit a lack of generalization to heterogeneous datasets, which may be due to limited training data or issues associated with overfitting. Interpretability of deep learning models is yet another issue as most existing approaches do not provide sufficient explainability for clinical decision-making. In addition, while classification and segmentation models perform well individually, not many works manage to effectively integrate both tasks into a unified and optimized framework.

To solve these problems, the present research presents a new deep learning method combining segmentation and classification for gastrointestinal disease diagnosis, enhancing both computational efficiency and generalization ability. The suggested GISegNet segmentation model has superior performance compared to current architectures with greater accuracy and efficiency. Meanwhile, the hybrid classification model utilizes transformer-based networks for improving feature extraction processes while minimizing computational expenses simultaneously. Further, the application of explainable AI methods improves the interpretability of the results, making them more clinically applicable.

## Materials and methods

This section offers an in-depth analysis of the datasets utilized in the training and testing phases of the developed models, as well as a discussion of the features of the specialized blocks in the model architecture and the methodologies employed.

### Dataset

**Fig. 1:**
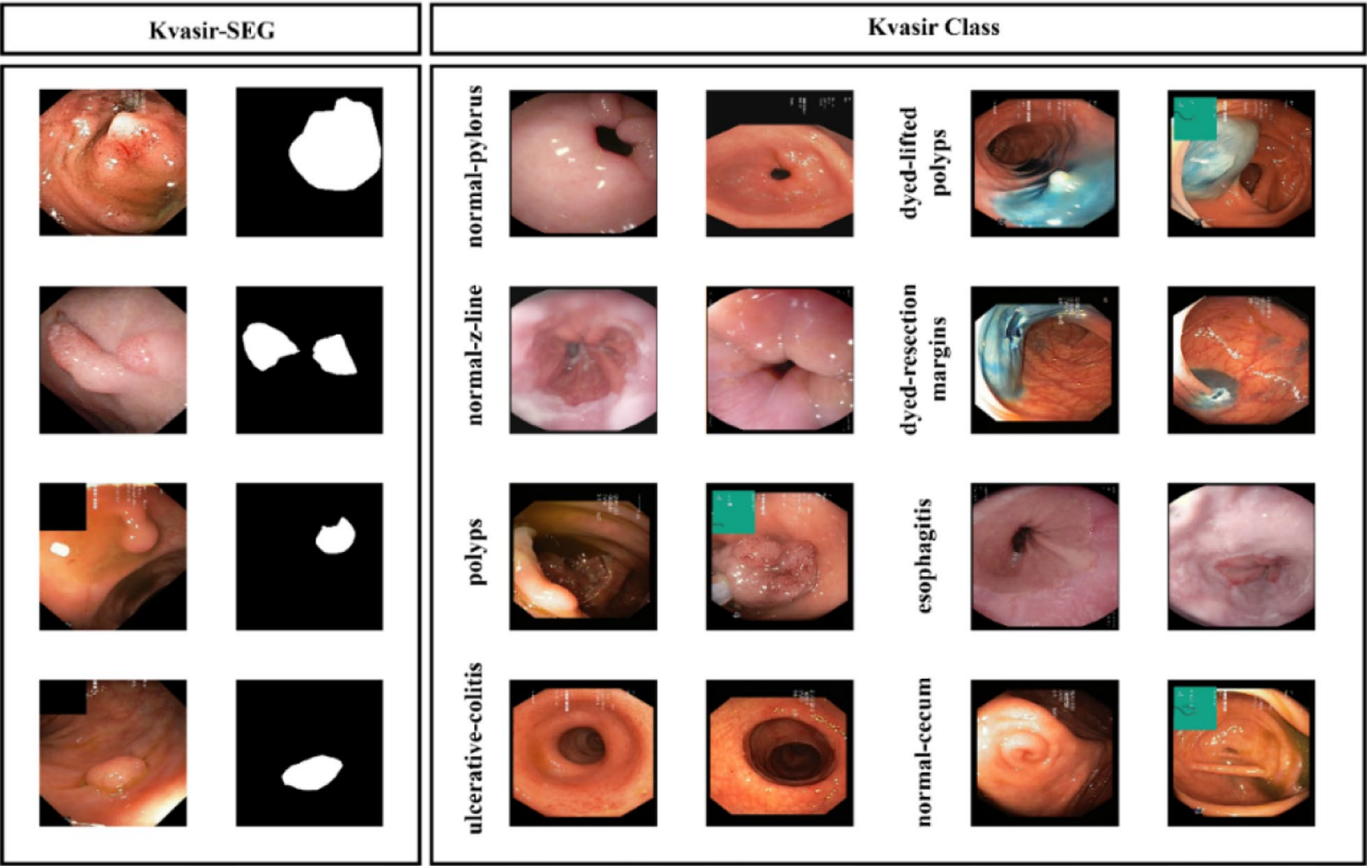
Sample images Kvasir dataset

In this study, the open-access Kvasir dataset was employed for training and evaluating the developed models. This dataset is organized into three primary anatomical landmarks and three clinically significant findings, along with two categories related to endoscopic polyp removal procedures. The dataset’s sorting and annotation were carried out by skilled endoscopists. Data collection was performed using endoscopic equipment at healthcare facilities affiliated with Vestre Viken Health Trust (VV) in Norway, which operates four hospitals and serves a population of approximately 470,000. The training data were sourced from Bærum Hospital, home to a large gastroenterology department. Expert annotators from VV and the Cancer Registry of Norway (CRN) meticulously labeled the images. The Kvasir dataset contains images that have been categorized and validated by experienced endoscopists, covering anatomical features, pathological conditions, and endoscopic procedures within the GI tract. The images, with resolutions ranging from 720 × 576 to 1920 × 1072 pixels, are organized into directories based on content. Additionally, the Kvasir-SEG dataset, derived from Kvasir v2, includes 1000 polyp images along with their corresponding ground truth masks. The dataset is 46.2 MB in size, with image resolutions varying from 332 × 487 to 1920 × 1072 pixels [22]. Example images from the Kvasir dataset are provided in Fig. 1. The dataset can be accessed through the following link: https://www.kaggle.com/datasets/abdallahwagih/kvasir-dataset-for-classification-and-segmentation

### Efficient strategies for optimizing convolutional operations

Convolutional Neural Networks (CNNs) are deep learning architectures widely used in areas such as image recognition and processing. These architectures are designed to extract diverse features from input images, enabling them to perform tasks such as classification, object detection, and segmentation. The fundamental operation of Convolutional Neural Networks (CNNs), convolution, can take several forms, such as standard convolution, depthwise convolution, and atrous (dilated) convolution. However, directly applying these convolution techniques can lead to an increase in the number of model parameters and computational complexity, presenting challenges in terms of optimization. In this study, to enhance the performance of convolution operations, standard, depthwise, and atrous convolutions have been adapted into asymmetric convolution techniques and applied more efficiently. The standard convolution operation can be described by the general formula shown in Eq. 1.

1 $$y\left(i,j\right)=\sum_{m=1}^{M}\sum_{n=1}^{N}x\left(i+m,j+n\right)*k(m,n)$$

In Eq. 1, $x(i,j)$ denote the pixel value of the input image at position $(i,j)$, while $k\left(m,n\right)$ represents the $M\times N$ filter kernel. The value at position $\left(i,j\right)$ of the resulting output feature map is denoted as $y\left(i,j\right)$, and $M,N$ specify the dimensions of the filter kernel.. While Eq. 1 represents the fundamental mathematical structure of convolution operations in CNNs, the asymmetric convolution techniques employed in this study aim to reduce computational complexity and enhance efficiency.

Asymmetric convolution offers an alternative approach to traditional convolution methods, aiming to significantly reduce the model’s parameter count and computational cost. This method involves decomposing a two-dimensional $n\times n$ convolution kernel into two sequential one-dimensional convolution operations. In the initial step, a vertical convolution kernel of size $n\times 1$ is applied, followed by a horizontal convolution kernel of size $n\times 1$ to complete the operation. This transformation allows the computation to be performed with only $2n$ parameters, compared to the ${n}^{2}$ parameters required when using a traditional $n\times n$ kernel. As a result, both memory consumption is reduced and computational cost is optimized. The mathematical expression of the asymmetric convolution operation can be defined as shown in Eq. 2.

2 $$y\left(i,j\right)=\sum_{m=1}^{M}\left[\sum_{n=1}^{N}x\left(i+m,j\right)*{k}_{v}(m)\right]*{k}_{h}(n)$$

In Eq. 2, $x(i,j)$ represents the pixel value at position $(i,j)$ of the input image, ${k}_{v}(m)$ denotes the vertical convolution kernel of size $n\times 1$, ${k}_{h}(n)$ is the horizontal convolution kernel of size $1\times n$, $y(i,j)$ represents the value at position $\left(i,j\right)$ of the output feature map, and $M,N$ refer to the kernel dimensions. Equation 2 clearly illustrates the operation structure of asymmetric convolution and the advantages it provides. Unlike traditional methods, this approach offers an optimization that enhances performance while reducing computational cost.

Atrous convolution, unlike traditional convolution methods, is a technique where spaced zero values are added within the convolution kernel. This method aims to increase the effective receptive field of the kernel while maintaining the number of pixels, thus improving computational efficiency. Atrous convolution can be expressed as shown in Eq. 3.

3 $$y\left(i,j\right)=\sum_{m=1}^{M}\sum_{n=1}^{N}x(i+r\cdot m,j+r\cdot n)\cdot k(m,n)$$

**Fig. 2:**
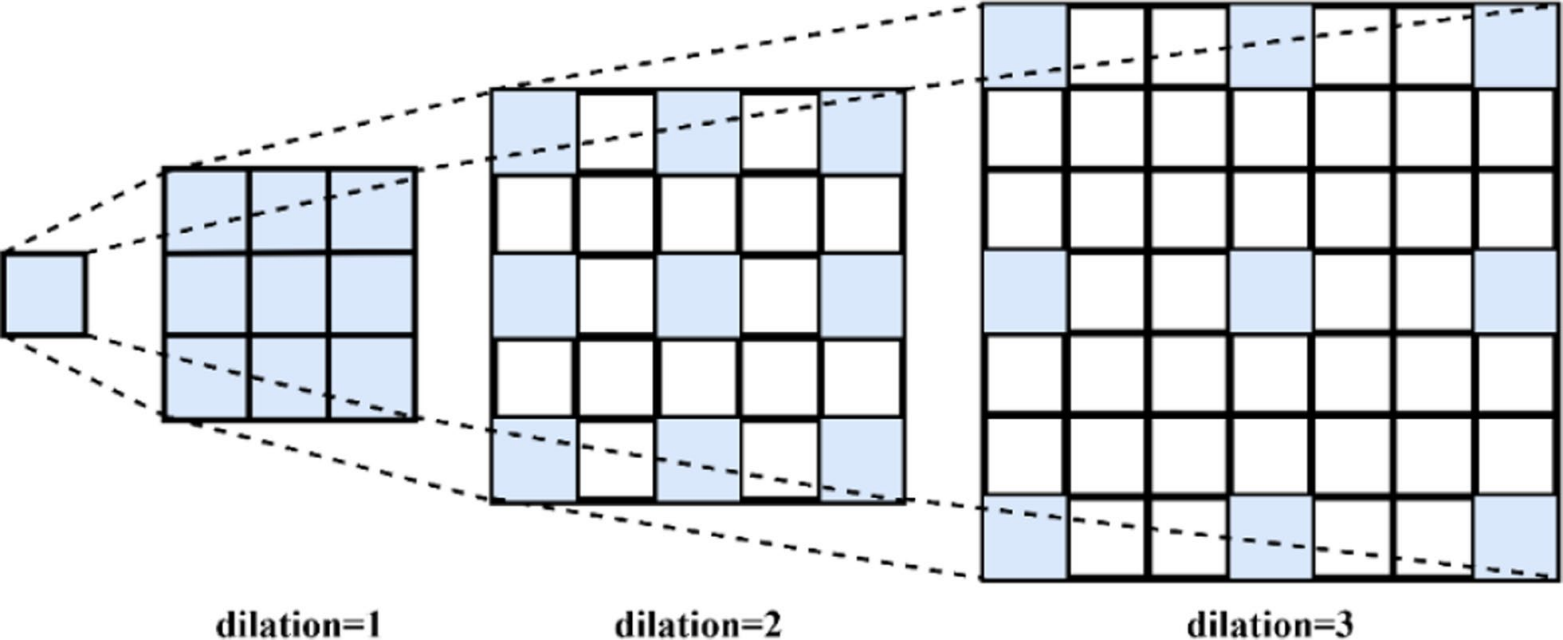
Illustration of convolution kernels with varying dilation rates

One of the key parameters used in atrous convolution is the dilation rate, which refers to the number of zeros added between consecutive elements of the kernel. When the dilation rate $r=1$, atrous convolution operates in the same way as standard convolution. However, when the dilation rate $r>1$, the receptive field of the kernel is expanded, allowing information to be gathered from a larger region. This enables the consideration of a broader contextual area without increasing the kernel size. Atrous convolution is particularly favored in tasks such as segmentation and other applications that require dense feature maps, as it provides a wider contextual awareness. Visual representations of convolution kernels with different dilation rates are shown in Fig. 2.

Atrous convolution is an effective technique that expands the spatial sensing capacity of the convolution kernel, maintaining resolution without additional parameter burden or computational cost. By using the dilation rate, this method achieves an enhanced contextual coverage, enabling the extraction of more complex and richer features. The extended effective receptive field of an $nxn$ convolution kernel with a dilation rate *a* is expressed as shown in Eq. 4.

4 $${K}_{nxn}=\left[1+\left(n-1\right)*a\right]*\left[1+\left(n-1\right)*a\right]$$

In Eq. 4, the dilation rate *a* determines the width of the gaps left between the pixels on both axes of the kernel. The kernels used in atrous convolution can be transformed into an asymmetric structure, resulting in two one-dimensional expanded kernels with different dilation rates along the $x$ and $y$ axes. These kernels are denoted as ${K}_{n\times 1}$ and ${K}_{1\times n}$, respectively. The mathematical expressions of the expanded receptive fields along the $x$ and $y$ directions are shown in Eqs. 5 and 6.

5 $${K}_{n\times 1}=\left[1+\left(n-1\right)*{a}_{x}\right]$$

6 $${K}_{1\times n}=\left[1+\left(n-1\right)*{a}_{y}\right]$$

In Eqs. 5 and 6${a}_{x}$ and ${a}_{y}$ represent the dilation rates along the $x$ and $y$ axes, respectively. This strategy aims to optimize the computational cost while expanding the receptive field of the kernels. The total spatial receptive field is determined by the product of the kernels through the sequential application of asymmetric kernels. This process allows for capturing more detailed features while providing an expanded contextual awareness. The total spatial receptive field is calculated as shown in Eq. 7.

7 $${K}_{n\times n}=\left[1+\left(n-1\right)*{a}_{x}\right]*\left[1+\left(n-1\right)*{a}_{y}\right]$$

In Eq. 7, the combined effect of the expanded kernels along the $x$ and $y$ axes is expressed, allowing the atrous convolution kernel to gather information from a larger area. This approach provides notable benefits in terms of enhanced contextual understanding and improved computational efficiency, especially in tasks like image segmentation and object recognition. In this research, the asymmetric atrous convolution technique is investigated as an alternative to conventional atrous convolution methods, aiming to reduce the number of parameters and enhance computational performance. Asymmetric atrous convolution optimizes processing times by expanding the receptive field while minimizing parameter usage. This approach provides significant benefits, especially during the processing of large and complex datasets, by optimizing memory usage and increasing process speed. Depthwise convolution is a method that processes multi-channel input data with kernels specifically defined for each channel. This method independently processes each input channel, enabling more efficient extraction of channel-specific features. Depthwise convolution is performed in a two-stage process: first, a convolution is applied along the x-axis with an $n\times 1$ kernel, and then the process is completed along the y-axis with a $1\times n$ kernel. As a result of this sequential process, an n × n receptive field is created. Asymmetric convolutions provide parameter efficiency compared to standard convolution operations. For example, when the kernel size is $k=3$, asymmetric convolutions use 33% fewer parameters than standard methods. This reduces the computational load significantly while improving processing speed and memory usage without any loss in performance. The asymmetric convolution strategy is implemented through the sequential application of two 1D convolutions. This method minimizes the overall number of parameters without compromising the model’s ability to learn, thereby enabling efficient high-performance inference in sophisticated models. Focused on parameter optimization, this method was developed to achieve improvements in memory savings and processing time, especially for deep learning-based tasks. The model developed in this study aims to reduce both computational cost and improve output quality by utilizing optimized asymmetric atrous convolution blocks.

### Efficient hybrid attentional atrous convolution (EHAAC) module

**Fig. 3:**
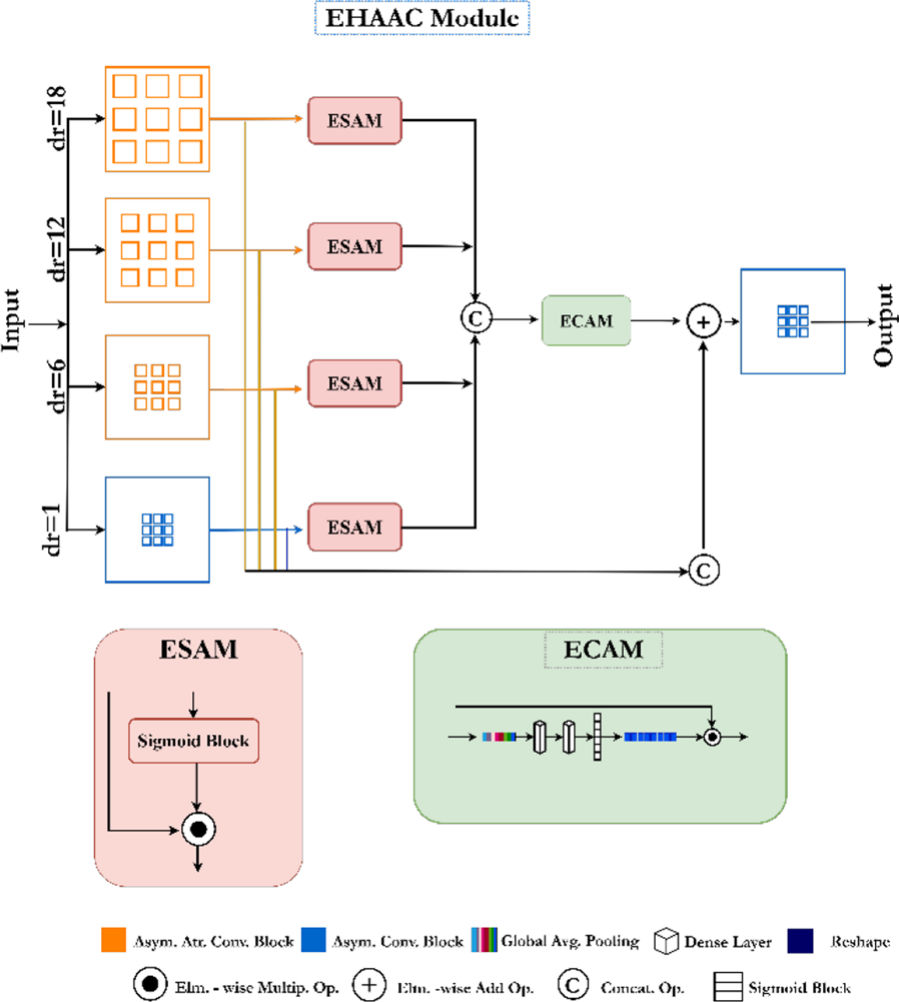
Structural components and functioning of the efficient hybrid attentional atrous convolution (EHAAC) module

Medical imaging technologies are critically important for the detection and segmentation of abnormalities in the human gastrointestinal (GI) system. This process plays a vital role in areas such as early diagnosis, treatment planning, and disease management. In response to these needs, the Efficient Hybrid Attentional Atrous Convolution (EHAAC) module has been developed to accurately detect abnormalities in endoscopic images of the GI system and optimize this process. EHAAC enhances contextual sensitivity, providing an effective tool for identifying and segmenting clinically significant findings such as polyps, ulcers, or inflammation. This module ensures clearer and more accurate delineation of target regions, thereby increasing diagnostic precision and facilitating better management of treatment processes. Furthermore, by combining expanded atrous convolution with attention mechanisms, it can efficiently analyze complex tissue structures. The structural components and functioning of the EHAAC module are detailed in Fig. 3. This structure aims to both improve accuracy in clinical applications and accelerate real-time diagnostic processes.

The integration of Atrous Convolutions with the Efficient Channel Attention Module (ECAM) seeks to enhance efficiency by detecting semantic features across a broader area in segmentation tasks, all while preserving resolution. This strategy aids in removing redundant information, thereby improving the network’s overall performance. Atrous convolution blocks process the input data with different dilation rates, allowing each block to extract features at various scales. The ECAM module is employed during this feature extraction process to filter the features based on their semantic importance. By processing feature maps with atrous convolutions at different dilation rates, this module enhances the semantic emphasis. This operation can be defined by Eq. 8.

8 $$ESAM\left(F\right)={\sum }_{i\in \{\text{1,6},\text{12,18}\}}\upsigma ({\updelta }^{i}\left(F\right))\odot {\updelta }^{i}\left(F\right)$$

The ECAM module focuses on the channel level, highlighting important features. Following the Global Average Pooling (GAP) operation, the mean value of each channel is transformed into a weight vector, facilitating more effective feature extraction. The processing flow of the ECAM module is expressed as shown in Eq. 9.

9 $$ECAM\left({F}_{ESAM}\right)={F}_{ESAM}\odot ({V}_{1x1}\odot\upsigma ({V}_{{w}^{2}}\odot\upsigma ({V}_{{w}^{1}}\odot {V}_{GAP}({F}_{ESAM}))))$$

Finally, the EHAAC module applies dilated convolutions on the feature map processed by the ECAM. This operation is expressed as shown in Eq. 10. This process improves the network’s efficiency and accuracy, leading to more accurate results in the segmentation process.

10 $$EHAAC\left(F\right)={\updelta }^{i}\left[{F}_{ECAM}+\sum_{i\in \{\text{1,6},\text{12,18}\}}\upsigma ({\updelta }^{i}\left(F\right))\odot {\updelta }^{i}\left(F\right)\right]$$

The combined structure shown in Eq. 10 provides an effective solution for high-accuracy and fast segmentation tasks, while also optimizing computational efficiency.

### Encoder decoder residual block

**Fig. 4:**
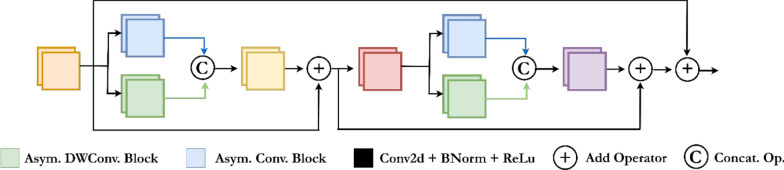
Schematic configuration of the encoder-decoder-residual (EDR) block

In this study, a neural network architecture has been developed to detect and segment abnormalities in the human gastrointestinal (GI) system. The proposed model is designed to identify abnormalities at the pixel level in images captured using remote sensing technology. It facilitates the efficient transmission of both local and contextual information, ensuring accurate classification of abnormalities. To optimize information flow, the Encoder-Decoder-Residual (EDR) block is incorporated into the network architecture, specifically tailored for abnormality detection. The EDR block features asymmetric convolution layers, allowing for deep and efficient processing of information. This design enhances the model’s ability to detect gastrointestinal abnormalities with greater precision and clarity. Figure 4 provides a detailed schematic of this innovative EDR block, which contributes to improving the model’s accuracy.

In the evaluation process outlined within the structural framework shown in Fig. 4, the input feature map *F* is first processed through a 1 × 1 convolutional layer, followed by batch normalization and *ReLU* activation, yielding ${F}_{{cbr}^{1}}$. This operation is mathematically represented by Eq. 11.

11 $${F}_{{cbr}^{1}}=ReLU(BN\left({Conv}_{1\times 1}\left(F\right)\right))$$

Subsequently, ${F}_{{cbr}^{1}}$ is processed by two different convolutional blocks (Asymmetric Convolutional Block (ACB) and Depthwise Asymmetric Convolutional Block (ADB)), resulting in ${F}_{{acb}^{1}}$ and ${F}_{{adb}^{1}}$, as expressed in Eqs. 12 and 13, respectively.

12 $${F}_{{acb}^{1}}=ACB\left({F}_{{cbr}^{1}}\right)$$

13 $${F}_{{adb}^{1}}=ADB({F}_{{cbr}^{1}})$$

The two feature maps expressed in Eqs. 12 and 13 are then combined to create ${F}_{combined}$, as shown in Eq. 14, for a richer flow of information.

14 $${F}_{combined}={F}_{{acb}^{1}}\oplus {F}_{{adb}^{1}}$$

The operation expressed in Eq. 14 is repeated to enhance the features and strengthen the flow, producing a second feature map, ${F}_{{add}^{2}}$, as expressed in Eq. 15.

15 $${F}_{{add}^{2}}=EDRBlock({F}_{{add}^{1}})$$

Finally, the obtained ${F}_{{add}^{2}}$ and ${F}_{{cbr}^{1}}$ are combined to enhance the performance of the deep learning model, resulting in the final output, ${F}_{finalOutput}$, as expressed in Eq. 16.

16 $${F}_{finalOutput}={F}_{{cbr}^{1}}+{F}_{{add}^{2}}$$

This approach guarantees that crucial information is retained in the deeper layers of the model, thereby enhancing its ability to detect abnormalities with higher accuracy.

### Proposed segmentation model (GISegNet)

**Fig. 5:**
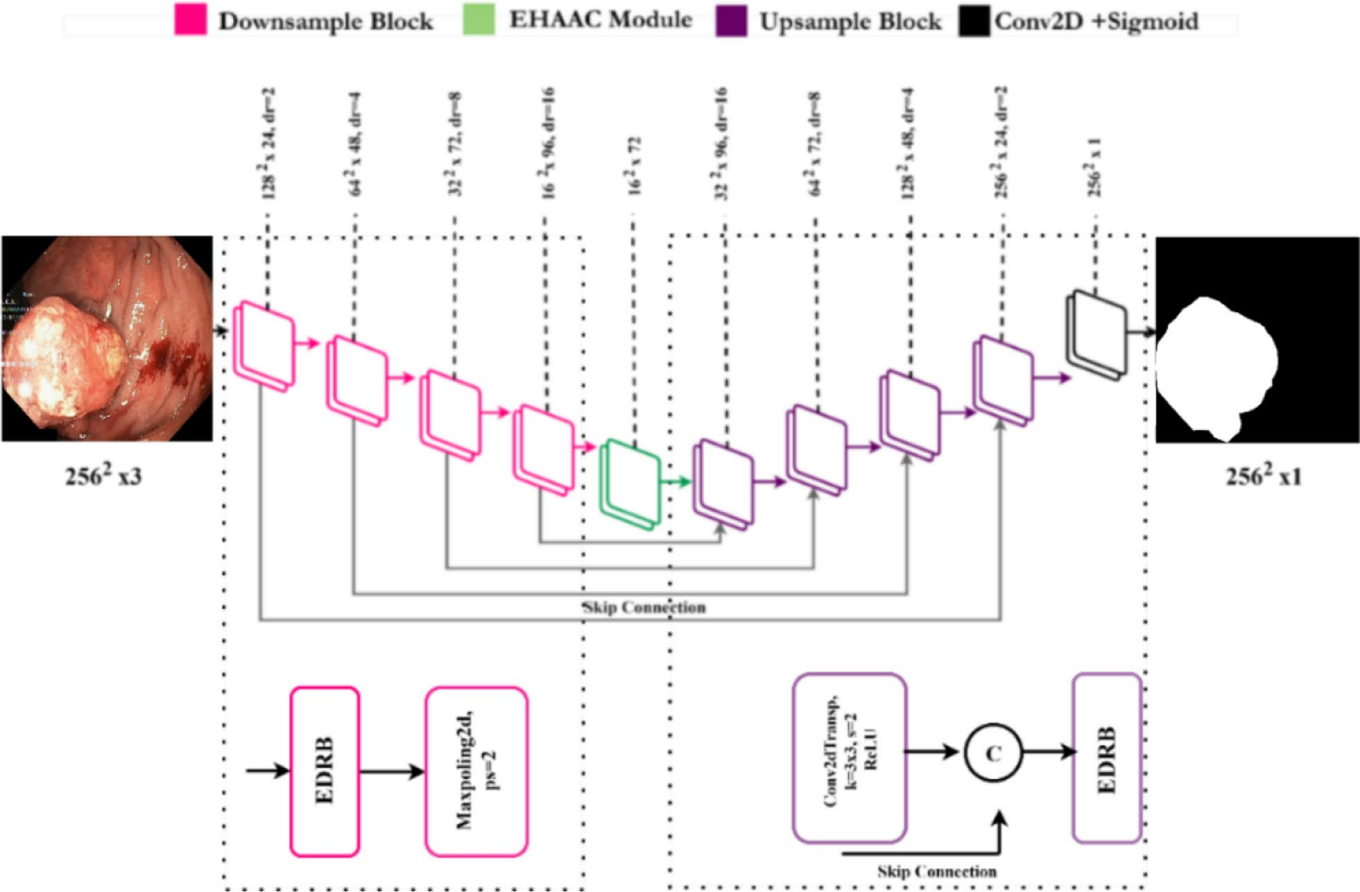
Overall architecture of the GISegNet segmentation model

This study introduces a novel convolutional neural network model, GISegNet, designed for the detection and segmentation of abnormalities in the human gastrointestinal (GI) system. The model comprises two primary components: the encoder and the decoder. The encoder includes sampling blocks and the Efficient Hybrid Attentional Atrous Convolution (EHAAC) module, which improves the model’s performance by integrating attention mechanisms with atrous convolution, thereby preserving both local and semantic information. The sampling blocks, in combination with Encoder-Decoder-Residual (EDR) blocks and max pooling layers, enable the model to detect even minor anomalies. The architecture of the GISegNet segmentation model is illustrated in Fig. 5.

The developed GISegNet model’s decoder section processes the input feature maps using 3 × 3 kernels and is then resampled with 2 × 2 stride transpose convolution layers. During this process, residual connections ensure the preservation and enhancement of features. The final feature maps are processed using 1 × 1 convolution and a sigmoid activation function, resulting in binary segmentation outputs. GISegNet is an effective model designed with an optimized structure to accurately detect abnormalities in the GI system.

### Data preparation process for classification

**Fig. 6:**
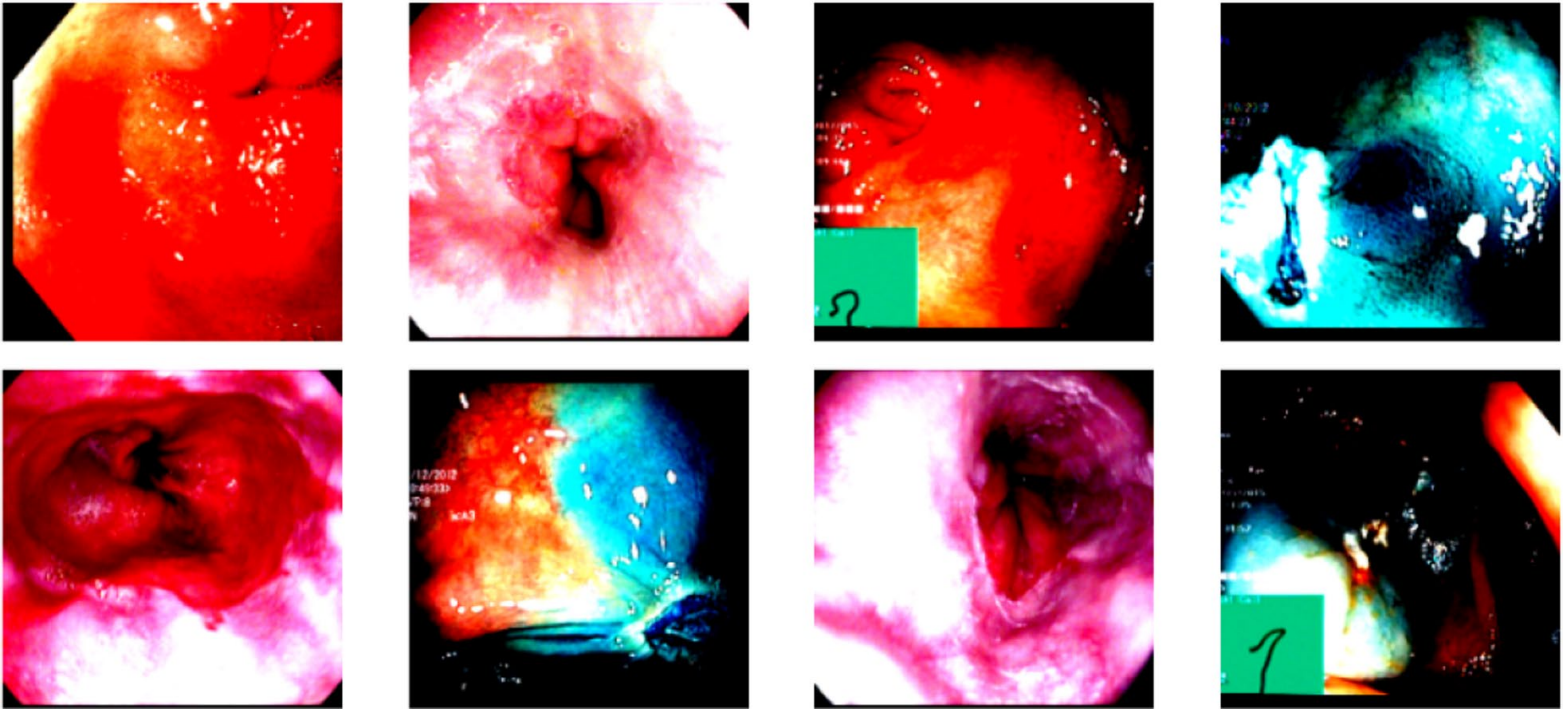
Sample dataset ımages following preprocessing steps

In the conducted study, an eight-class gastrointestinal (GI) system dataset was used. The images were preprocessed to a resolution of 224 × 224 pixels. Data augmentation techniques, such as RandomResizedCrop and RandomHorizontalFlip, were applied during training, while basic scaling operations were performed during testing and validation. The dataset was divided into 60% for training, 20% for validation, and 20% for testing purposes. Figure 6 presents example images from the dataset following the preprocessing steps.

### Support vector machines

**Fig. 7:**
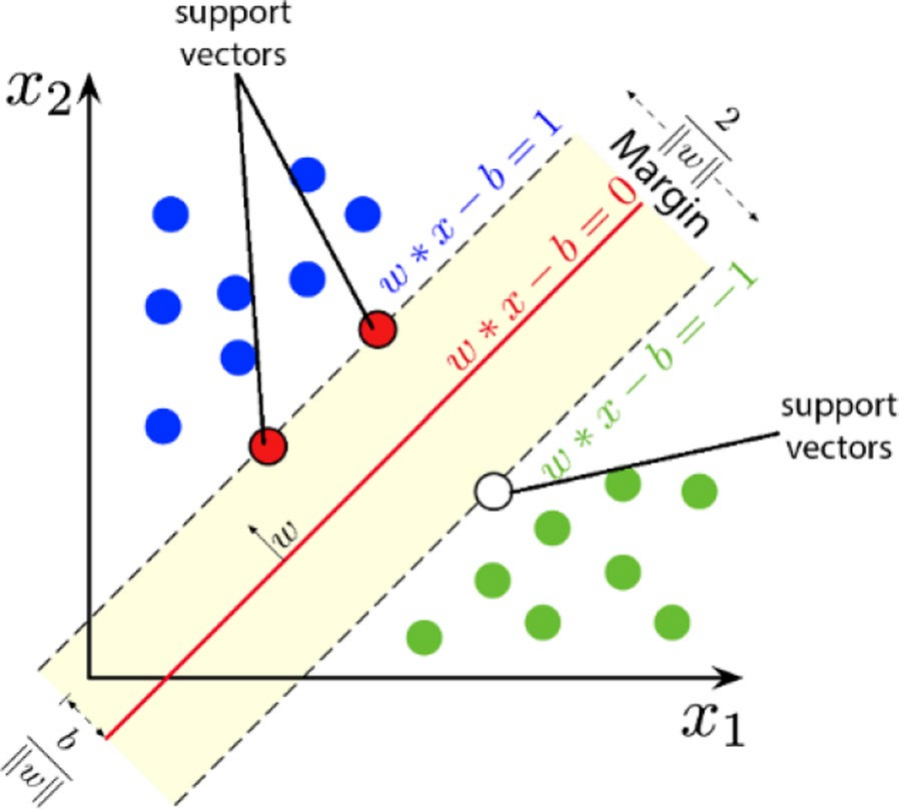
Support vector machines classification example

Support Vector Machines (SVM) are an effective supervised learning method aimed at classifying data points by determining a hyperplane that separates different classes in classification problems. SVM is particularly successful with small to medium-sized, complex datasets [23]. The fundamental principle of the model is to create a decision boundary that maximizes the distance (margin) between classes. In Fig. 7, the classification process with SVM is visualized, where the black and white points represent different classes, and the pink area represents the margin region at a distance of ± 1 from the hyperplane. A wider margin leads to better model performance.

In the SVM shown in Fig. 7, classification is performed using the weight vector $w$, input vector$x$, and bias$b$. If the computed value is less than 0, the sample is assigned to the green class, and if it is greater than or equal to 0, the sample is assigned to the blue class. This process is expressed by Eq. 17 [24].

17 $$y=\left\{\begin{array}{c}0\,if\, {w}^{T}.x+b<0,\\ 1\, if\, {w}^{T}.x+b\ge 0\end{array}\right.$$

In the hybrid model proposed for classifying abnormalities in the GI system, the SVM method uses a Cubic kernel function, with the Kernel scale set to Auto. The Box constraint level is chosen as 1, and the Multiclass method is implemented using the One-vs-One approach. These parameters have been appropriately configured to optimize the performance of the model.

### Feature selection approach: mRMR technique

Feature selection techniques are employed to enhance the classification performance of deep learning models. These methods focus on improving the model’s accuracy by identifying the most relevant features. In this research, the Minimum Redundancy Maximum Relevance (mRMR) method was selected for binary classification [25]. mRMR is a filtering technique used to ensure the highest correlation and minimum redundancy among features. The algorithm ranks each feature and evaluates their relationships, defining less important features as “redundant” and significant ones as “relevant.” This process was carried out using MATLAB’s Feature Selection Methods tool.

### Transformer models

**Fig. 8:**
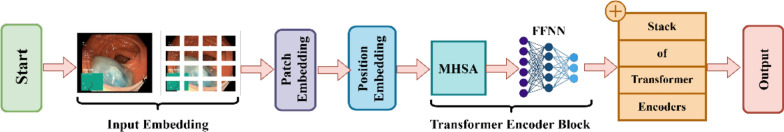
Processing stages of transformer models

Recent advancements in image analysis have been driven by deep learning models, especially Convolutional Neural Networks (CNNs) and Vision Transformers (ViTs). ViT models, by processing long-range dependencies more effectively, can achieve higher performance in the classification of abnormalities in the human gastrointestinal (GI) system. In this study, Deit3, Maxvit, Swin, and ViT models were used, with all images processed at a resolution of 224 × 224 pixels. The models’ architecture is based on six main processing stages, which may include additional layers. A visual representation of these processing stages is presented in Fig. 8.

In the ViT architecture shown in Fig. 8, the input images are processed by dividing them into patches. These patches, which are of size 16 × 16 or 24 × 24 pixels, are passed through an embedding layer to be converted into vector representations. The order of the patches is determined through positional embedding. The Transformer Encoder Blocks incorporate multi-head self-attention (MHSA) and feed-forward neural network (FFNN) components, enabling the model to capture more complex features. The output layer at the end of the model is responsible for performing the classification task. In this study, the following models with an input resolution of 224 × 224 pixels were utilized: 1 st DeiT3 Base Patch16-224, MaxViT Base-tf-224, Swin Base Patch4 Window7-224, and ViT Base Patch16-224. These models demonstrate notable performance in visual data analysis.

### Proposed hybrid approach for classification

**Fig. 9:**
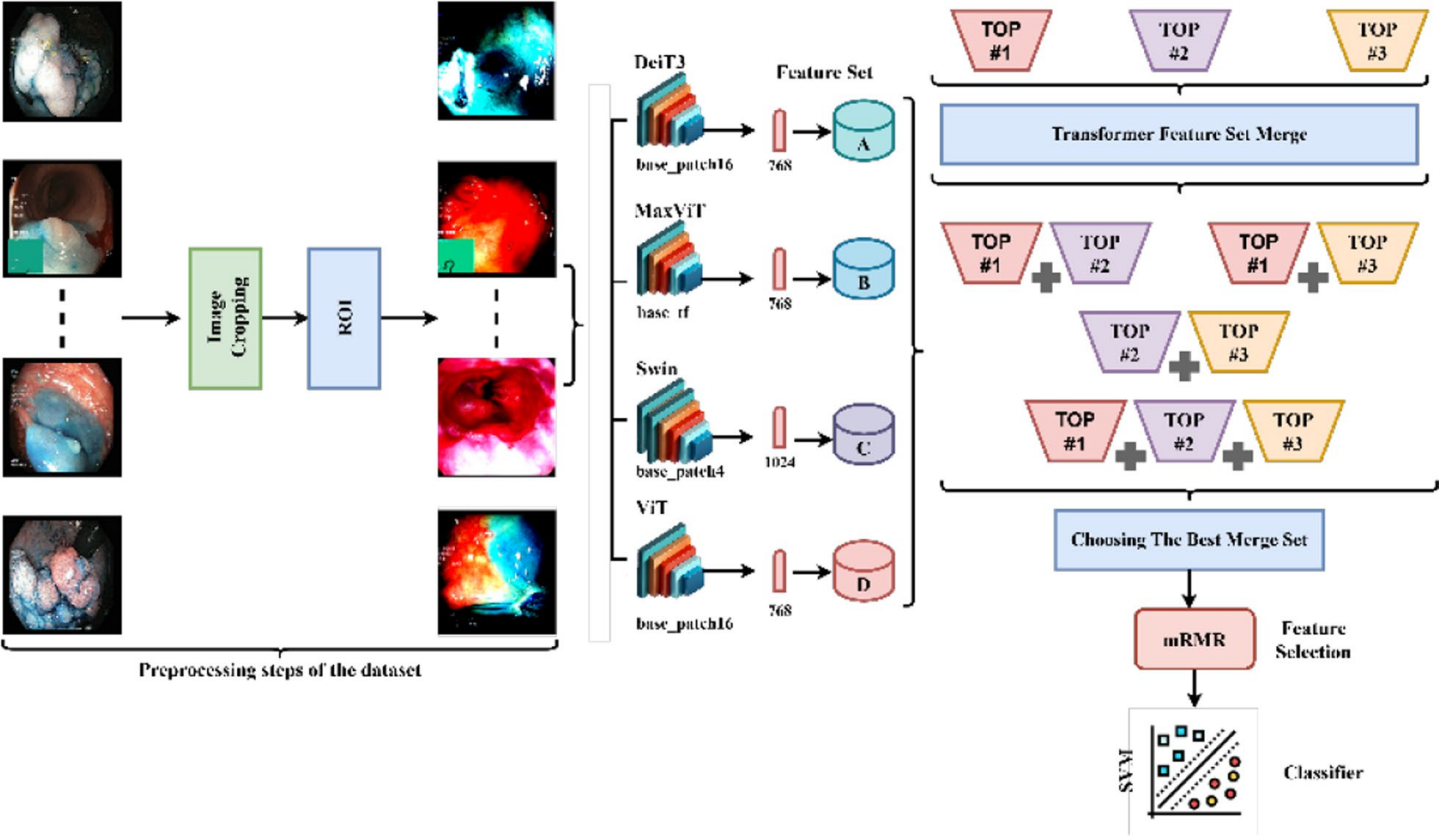
Overall architecture of the proposed hybrid model

The hybrid model proposed in this study aims for the fast and accurate analysis of human gastrointestinal (GI) images. Vision Transformer (ViT) models are central to this framework due to their efficiency in image processing and feature extraction. The model follows six primary stages: preprocessing, model training, feature extraction, feature fusion, feature selection, and classification. During the preprocessing phase, gastrointestinal (GI) images are enhanced, cropped, and resized to 224 × 224 pixels to provide ViT models with data that is both efficient and suitable for processing. In the training phase, models such as DeiT3/base patch16, MaxViT/base-tf, Swin/base patch4, and ViT base patch16 are employed to ensure the rapid and precise analysis of human GI images. These models can operate with low hardware requirements due to their simple architectures and low parameter counts. By using different ViT models, the feature sets obtained from GI images were diversified, and each model learned important features for classifying GI abnormalities with 224 × 224 pixel resolution input images. This approach ensures more efficient analysis of the data. During the feature extraction phase, features are extracted from the fully connected (FC) layer, prior to the classification layer, of each model. The DeiT3, MaxViT, and ViT models each produce a feature set of 768, whereas the Swin model generates 1024 features due to its unique architecture. The models’ performance was assessed using Support Vector Machine (SVM), and the feature sets from the top three models were identified. These sets were subsequently combined for further analysis, leading to the creation of four new feature sets derived from the union of the DeiT3, Swin, and ViT models. The performance of these combined feature sets was also evaluated using SVM. In the feature selection phase, the best-performing set among the combined sets was selected for subsequent steps. The selection was conducted using the Minimum Redundancy Maximum Relevance (mRMR) method, and new sets with 100, 300, 500, 700, and 1000 features were generated from the chosen set. These new sets were then classified using the SVM method. The overall architecture and processing steps of the proposed hybrid model are visually depicted in Fig. 9.

## Experimental results and discussion

**Fig. 10:**
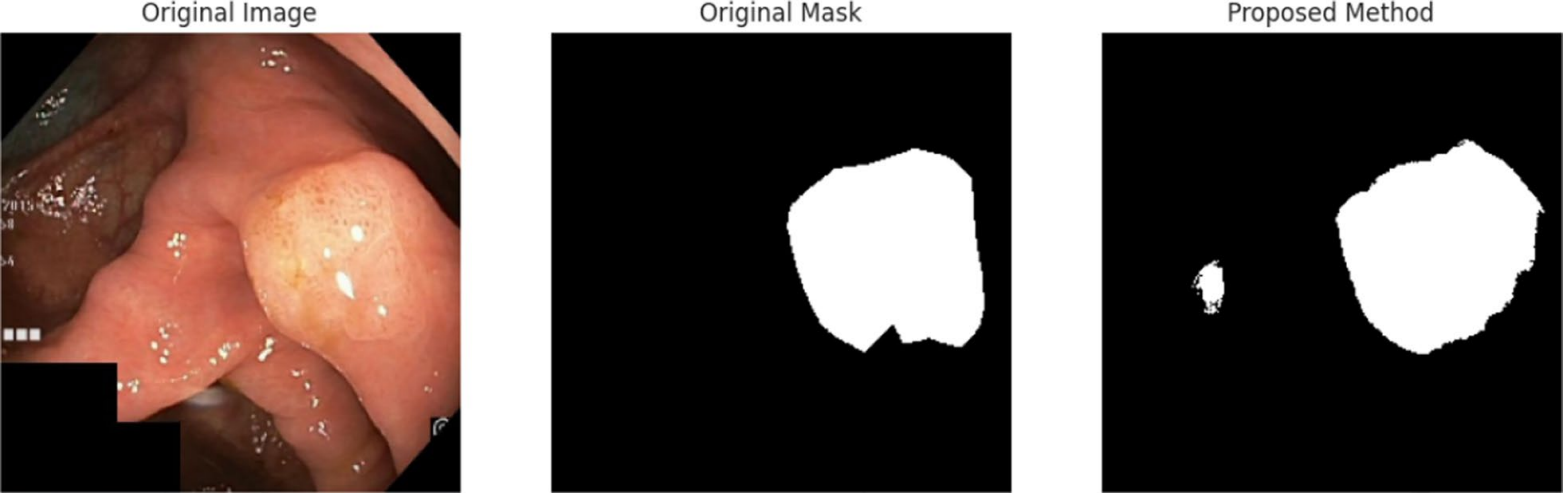
Visualization of the mask results generated after the training phase of the proposed method

In the initial phase of this study, the focus was on detecting and segmenting abnormalities in the human gastrointestinal (GI) system. The proposed approach utilizes a specialized convolutional neural network model, GISegNet, which was trained and tested using the Kvasir dataset, with an emphasis on semantic segmentation tasks. The performance of GISegNet was compared to existing segmentation models, such as UNet and PSPNet. The performance evaluation involved several steps. First, images and their corresponding masks were loaded into the system. Then, these images and masks were visualized to aid in enhancing the model’s effectiveness. For data preprocessing, the images were prepared for both training and evaluation, with the dataset divided into 80% for training, 10% for testing, and 10% for validation, while the input image size was standardized to 256 × 256 pixels across all models. The model was then trained using the training data for 100 epochs. After training, the model made mask predictions on the test data, and the resulting predictions were visualized. Figure 10 shows the predicted mask image obtained after the model’s testing phase.

The GISegNet model was trained on the Google Colab platform using Python, TensorFlow, and Keras. For optimization, the Adam optimizer was employed, and the learning rate was adjusted using the Keras ReduceLROnPlateau function. The initial learning rate was set at 0.01, with a minimum learning rate of 0.00001 and a batch size of 16. The model underwent training for a total of 100 epochs. To assess the model’s performance, several metrics were used, including Accuracy, Precision, Recall, Dice Similarity Score (DSS), and Mean Intersection over Union (mIoU). These metrics were calculated based on true positive (TP), false positive (FP), true negative (TN), and false negative (FN) values, as outlined in Eqs. 18–23.

18 $$Acc=\frac{TP+TN}{TP+TN+FP+FN}$$

19 $$Prec = TP/(TP+FP)$$

20 $$Rec = TP/(TP+FN)$$

21 $$F1-Scr=\frac{2\times TP}{2\times TP+FP+FN}$$

22 $$Dice SS = \frac{2.Prec \times Rec}{Prec+Rec}$$

23 $$mIoU=\frac{TP}{TP+FP+FN}$$

**Fig. 11:**
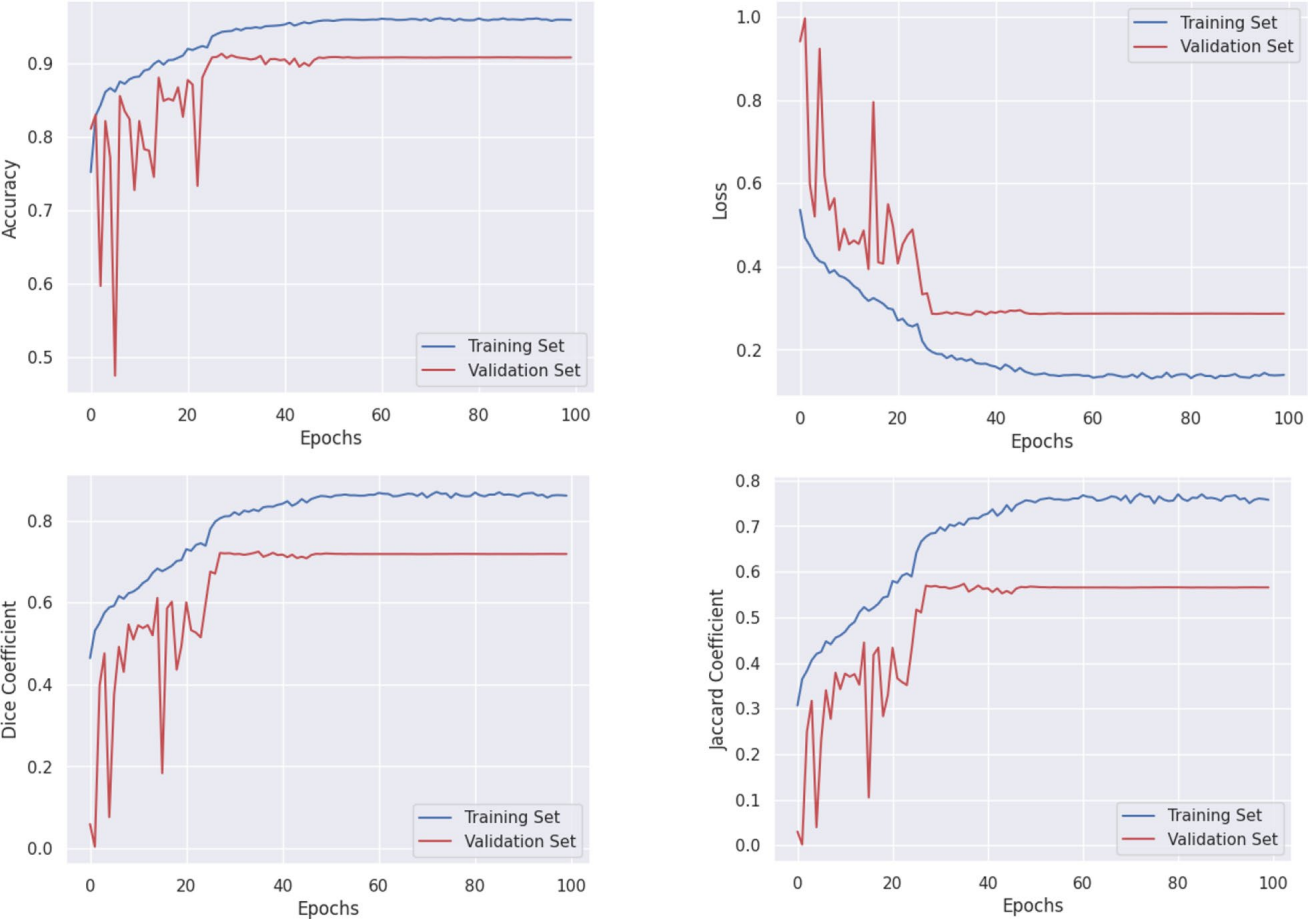
Graphs of accuracy, loss, dice, and Jaccard coefficients for the GISegNet model on the Kvasir dataset

Figure 11 presents the accuracy, loss, Dice coefficient, and Jaccard index graphs for the training process of the GISegNet model on the Kvasir dataset. During the training process, the model’s loss initially started at 0.5359 and decreased to 0.1393 by the end. This indicates significant improvement during training, with the loss value steadily decreasing. The validation loss, which started at 0.9407, decreased to 0.2863, suggesting that the model showed better performance on the validation data over time. The Jaccard index started at 0.3064 during training and increased to 0.7576 by the 100 th epoch. This shows a significant improvement in the model’s ability to predict overlapping regions during training. The validation Jaccard index, which began at 0.0303, increased to 0.5655, indicating improved overlap during the validation process. The Dice coefficient started at 0.4640 and increased to 0.8606 by the 100 th epoch, demonstrating a significant improvement in the model’s ability to detect positive classes. The validation Dice coefficient rose from 0.0588 to 0.7183, indicating better recognition of positive classes on the validation data. The accuracy during the training process initially was 75.17%, reaching 95.87% by the end, while the validation accuracy increased from 81.05 to 90.77%. These high accuracy rates suggest that the model made highly accurate predictions overall with a very low error rate. In conclusion, the model showed significant development throughout the training process and achieved excellent results on the validation data, demonstrating its high generalization ability and successful performance on different datasets.

**Fig. 12:**
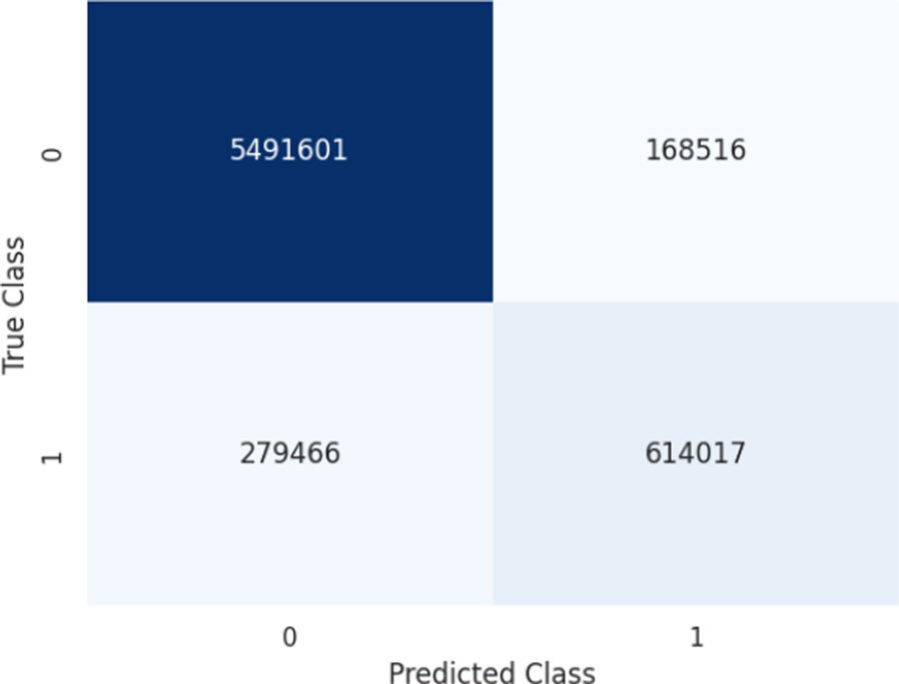
Confusion matrix of GISegNet model

After completing the training and testing phases, the strengths of the GISegNet model were thoroughly evaluated. The similar loss and accuracy values achieved during both the training and validation phases indicate the model’s ability to generalize effectively to new and unseen data. The Dice and Jaccard coefficients obtained during training reflect the model’s high precision and sensitivity at the pixel level. These characteristics are crucial for detecting abnormalities in the human gastrointestinal (GI) system, as early detection is essential for selecting appropriate treatments and expediting patient care. Thus, the model’s ability to produce precise results is essential for accurate disease diagnosis and enables timely intervention. The stable and low loss values throughout training further suggest that the learning process was consistent, and the model made steady improvements. This stability indicates that the model can maintain its performance across prolonged training sessions and large datasets. The high accuracy rate highlights the model’s capacity to detect diseases rapidly and reliably, providing a considerable advantage in clinical environments. The confusion matrix for the developed model, after training with the Kvasir dataset, is shown in Fig. 12.

In this study, the performance of the GISegNet model was benchmarked against several state-of-the-art models commonly employed in segmentation tasks. During the experimental phase, GISegNet, along with well-established models such as UNet and PSPNet, were trained and tested under identical conditions without utilizing transfer learning techniques. These models were chosen to provide a fair and unbiased comparison of GISegNet’s capabilities. All models were evaluated on the same datasets to ensure consistency in the performance analysis. Compared to existing segmentation models such as UNet and PSPNet, GISegNet introduces the Encoder-Decoder Redundancy (EDR) block and the Efficient Hybrid Attentional Atrous Convolution (EHAAC) mechanism, which enhance segmentation accuracy by capturing fine details and contextual relationships more effectively. The results of the performance evaluation for models tested on the Kvasir dataset are summarized in Table 2.

**Table 2:** Comparison of performance evaluation metrics of GISegNet and other segmentation models tested on the Kvasir dataset

Models	Input Size	Prec. (%)	Rec. (%)	Acc. (%)	mIoU (%)	DSS (%)	Total Params (M)	Training Time (Min)
UNet	256 × 256 × 3	57.00	62.91	88.47	65.03	59.81	31 055 297	74.08
PSPNet		70.60	64.18	91.47	70.65	67.24	579 713	16.13
**GISegNet (our)**		**78.46**	**68.72**	**93.16**	**75.13**	**73.27**	648 457	561.24

Upon examining Table 2, it can be observed that the GISegNet model demonstrates a significant superiority over the UNet and PSPNet models in terms of accuracy (%93.16) and other segmentation performance indicators. Specifically, the higher values achieved by GISegNet in metrics such as Precision (%78.46), Recall (%68.72), mIoU (%75.13), and DSS (%73.27) indicate that the model provides more reliable results in terms of correct classification and disease detection. These results suggest that GISegNet could be an important tool for the early detection of abnormalities in critical areas such as the human gastrointestinal (GI) system. However, it is noted that the total number of parameters in GISegNet (648,457) and the training time (561.24 min) are significantly higher compared to the other models. This indicates that the model requires a more complex structure and longer training time. Nevertheless, it should be emphasized that in critical applications such as disease diagnosis, the accuracy of the model should take precedence over training time. High accuracy provides greater reliability in making correct diagnoses and plays a significant role in medical processes, regardless of the model’s speed. Especially for early diagnosis and determining the right treatment strategies, high accuracy rates are crucial.

**Fig. 13:**
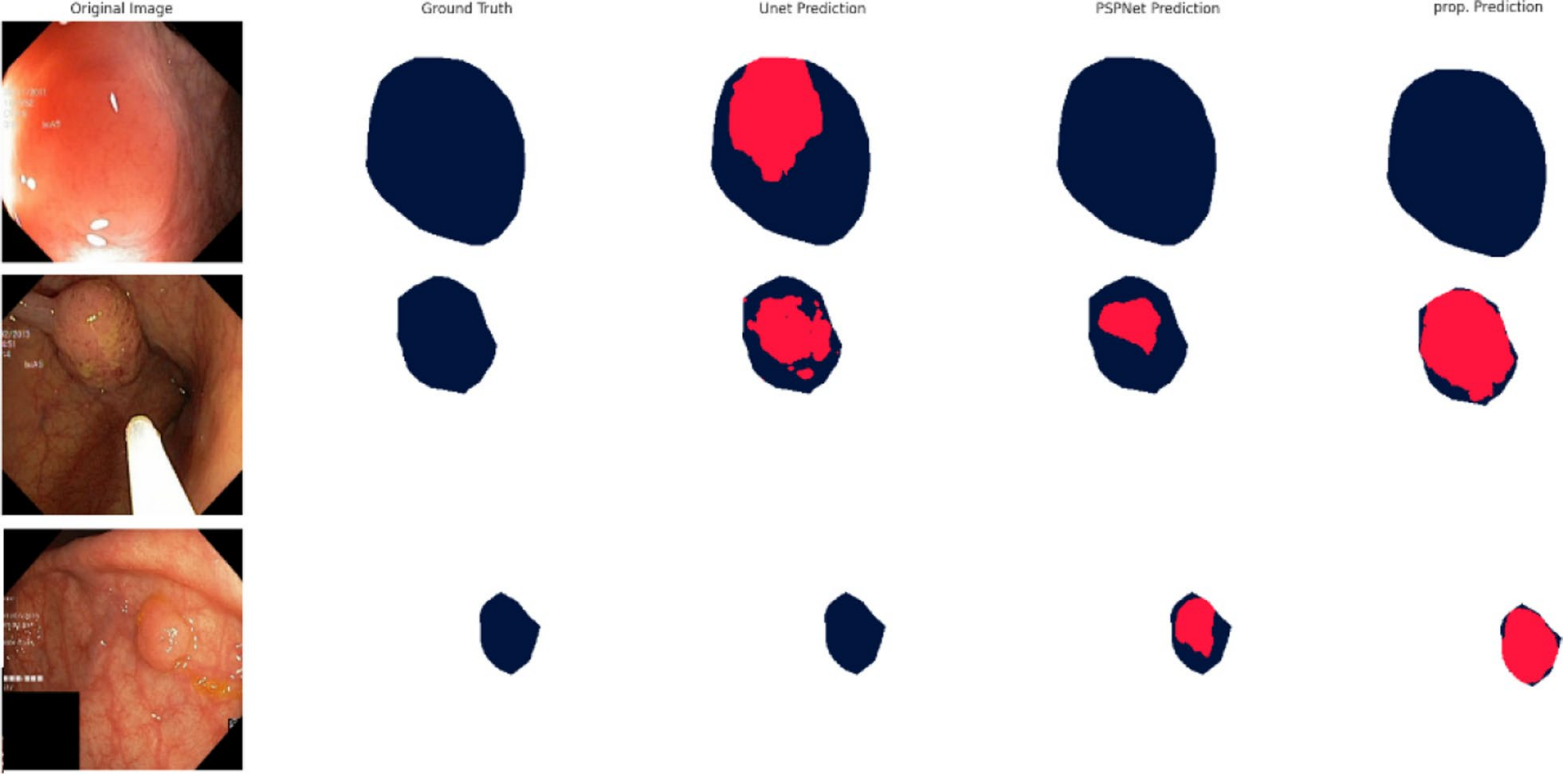
Visual comparison of GISegNet and other segmentation models tested with the Kvasirdataset

The performance of GISegNet, alongside other segmentation models, in delineating abnormal regions in the human gastrointestinal (GI) system using the Kvasir dataset is illustrated in Fig. 13. The first column displays the original images in three-channel RGB format, while the second column presents the manually annotated ground truth boundaries for the regions of interest. Subsequent columns showcase the prediction results from GISegNet and other segmentation models. Each column provides a visual comparison between the true and predicted boundaries, with the actual boundaries marked in blue and the predicted ones in red. The analysis indicates that GISegNet consistently identifies the boundaries of abnormal regions with greater clarity and precision compared to the other models. While GISegNet does encounter some difficulty in accurately defining boundaries in certain instances, it generally produces results with high accuracy and outperforms the other models in detecting disease. These visual comparisons serve as a critical tool for assessing the effectiveness of segmentation models in disease detection, providing valuable insight into their performance.

In the second phase of the study, a hybrid transfer learning model was developed to classify the images from the 8-class Kvasir dataset. The model development and testing were carried out using Python, TensorFlow, and Keras on the Google Colab platform. During the analysis process, feature fusion and feature selection methods were applied, and classification was performed using machine learning algorithms. MATLAB 2024 software was also used as a supportive tool during this process. A widely used method for evaluating classification accuracy, the confusion matrix, was one of the key tools used to validate the analyses conducted in the study.

The parameters of the Vision Transformer (ViT) models used in the study and their corresponding values were examined in detail. Experimental analyses based on the proposed method were conducted in three phases. Cross-entropy was chosen as the loss function, and the learning rate was set to 1 × 10^−5^. The optimization process used the SGD method, and the classifier had a linear structure. The training process was limited to 20 epochs, and the mini-batch size was selected as 2. The training and testing ratio was set to 0.8:0.2. These parameters aim to optimize the study in terms of accuracy and performance.

**Fig. 14:**
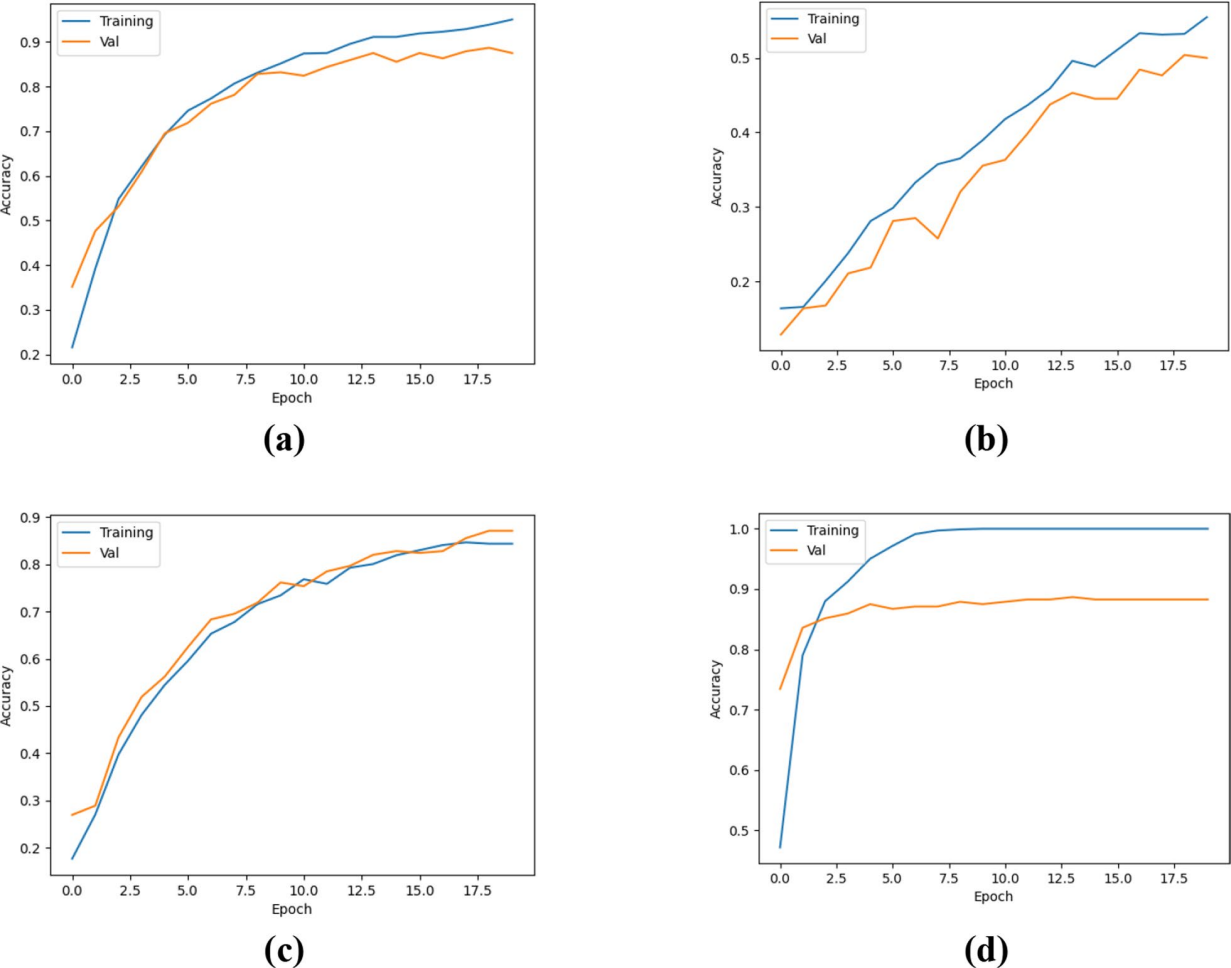
Classification performances of ViT models: **a** Deit3/base patch16, **b** MaxViT/base-tf, **c** Swin/base patch4, **d** ViT/base patch16

**Fig. 15:**
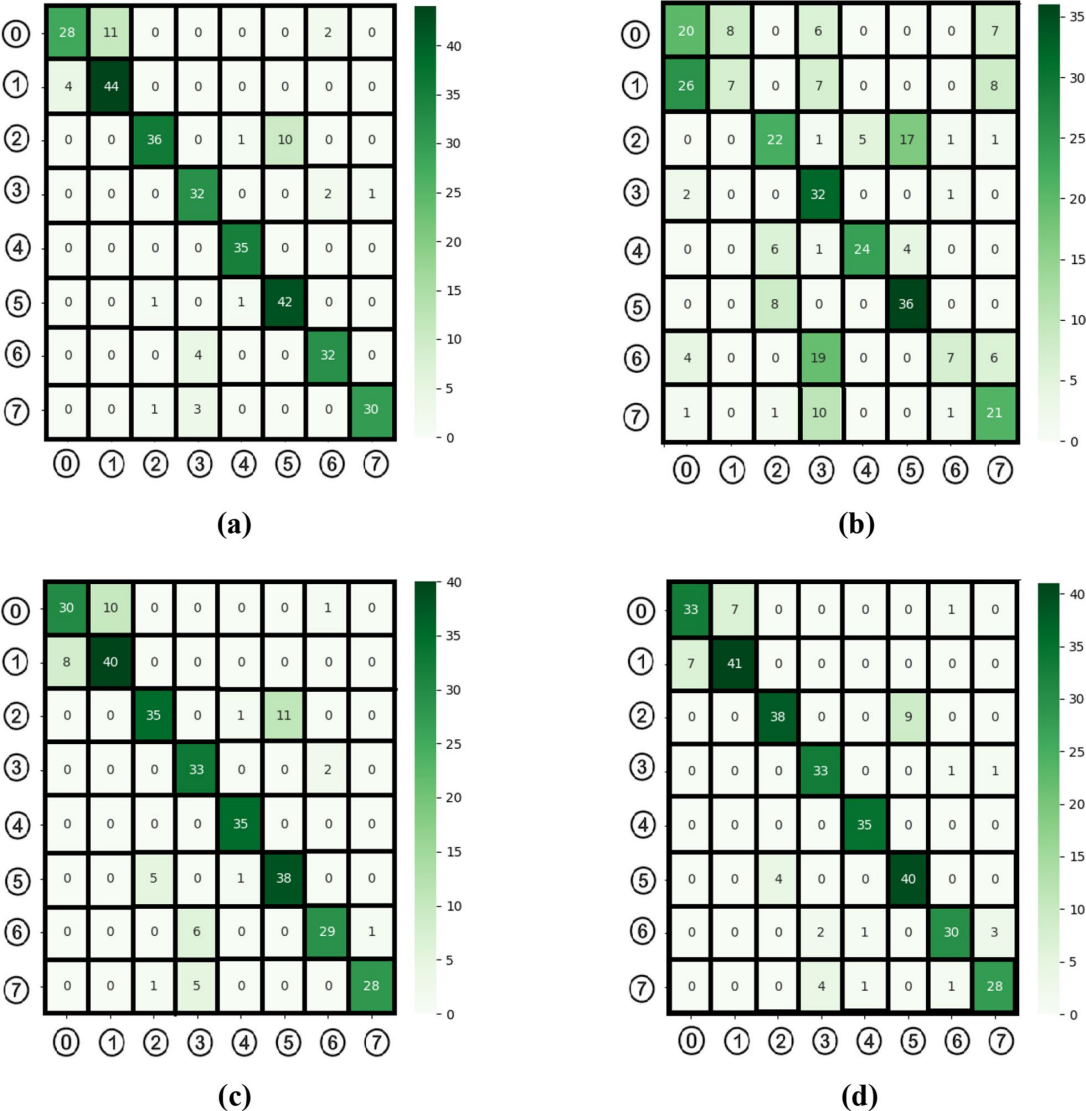
Confusion matrices obtained in the classification process of transformer models **a** Deit3/base patch16, **b** MaxViT/base-tf, **c** Swin/base patch4, **d** ViT/base patch16, classes 0: dyed-lifted-polyps, 1: dyed-resection-margins, 2: esophagitis, 3: normal-cecum, 4: normal-pylorus, 5: normal-z-line, 6: polyps, 7: ulcerative-colitis

Before beginning the classification process, the first step in making the dataset more efficient involved performing appropriate enhancement operations on the images. In the Python environment, the ‘ResizedCrop(224)’ function was used to resize the images to 224 × 224 pixels, centered. Then, the ROI (Region of Interest) method, developed with the support of the Pandas and Numpy libraries, was used to highlight the relevant areas. In the second phase of the study, the pre-processed data were used to train Transformer models, including DeiT3/base patch16, MaxViT/base-tf, Swin/base patch4, and ViT/base patch16. The data was split into 80% for training and 20% for testing, with a linear layer applied for the classification task. The performance of the models in terms of accuracy is depicted in Fig. 14, while the confusion matrices are presented in Fig. 15. The detailed results derived from these matrices are provided in Table 3.

**Table 3:** Metric results from the confusion matrix of transformer models (%)

Transformer model	Features	Recall	Pre	F1-Scr	Acc
Deit3/base patch16	768	87.19	87.83	87.02	87.19
MaxViT/base-tf	768	52.81	55.97	50.01	52.82
Swin/base patch4	1024	83.75	84.32	83.71	83.75
ViT/base patch16	768	87.19	87.83	87.02	87.19

According to the data in Table 3, the DeiT3 and ViT models achieved the highest performance with an accuracy rate of 87.19%. The Swin model ranked third with an accuracy of 83.75%, while the MaxViT model demonstrated lower performance with an accuracy rate of 52.82%. Despite the increase in time costs, the proposed method aims to improve classification success by merging feature sets obtained from different ViT models. In the subsequent phase, feature sets derived from the top three models were merged to improve performance. The DeiT3 and ViT models each produced 768 features, while the Swin model generated 1024 features. These feature sets were combined in various ways, resulting in several new sets: the DeiT3-Swin and ViT-Swin combinations contained 1792 features, the DeiT3-ViT combination contained 1536 features, and the DeiT3-ViT-Swin combination included 2560 features. The influence of these merged feature sets on classification performance was evaluated, and the findings are summarized in Table 4.

**Table 4:** Feature count and letter representations of combined models

Representing letter symbol	Models with combined features	Total number of features
I	DeiT3 and Swin*	1792
II	DeiT3 and ViT*	1536
III	Swin and ViT*	1792
IV	DeiT3 and Swin and ViT*	2560

**Fig. 16:**
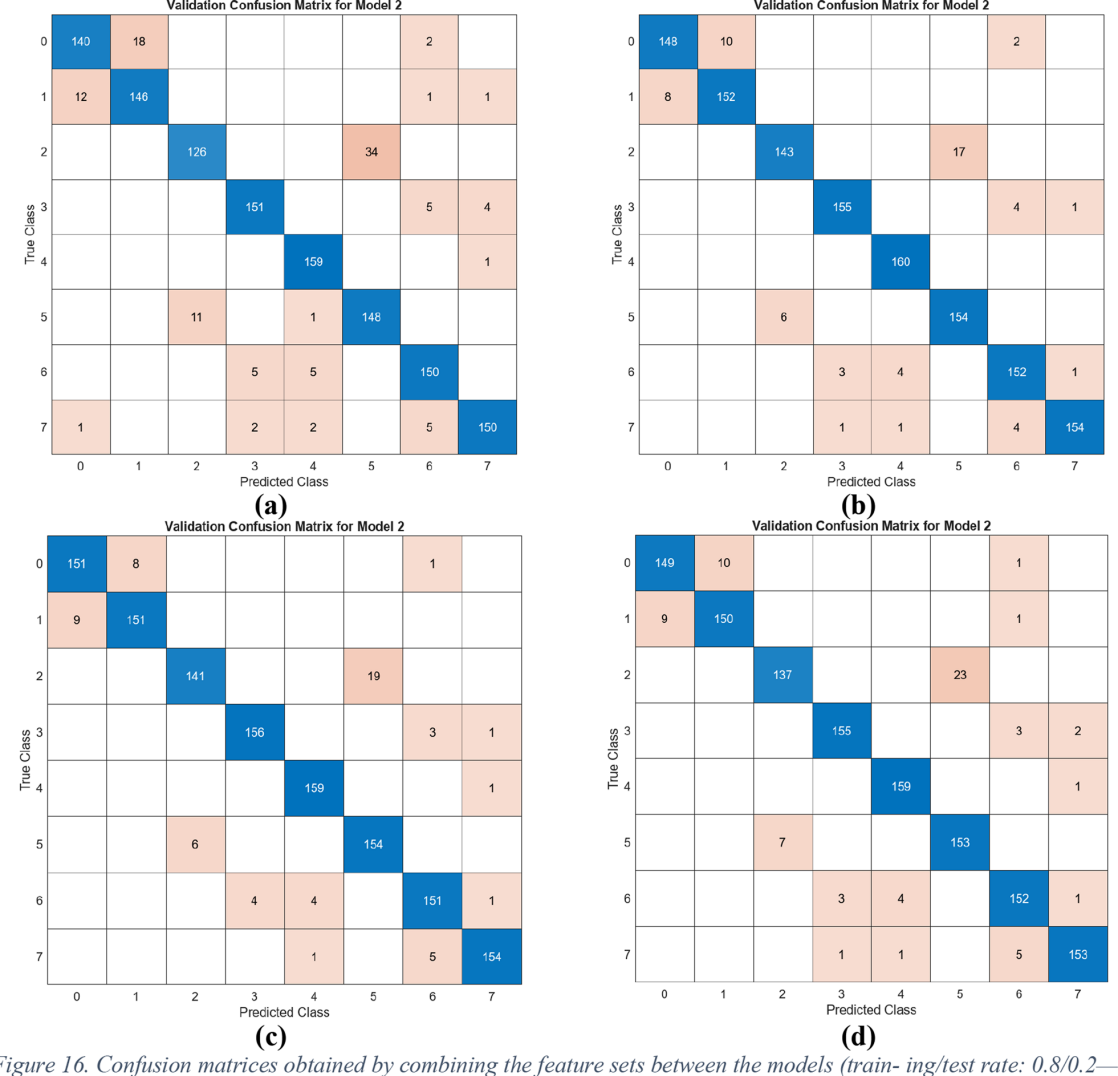
Confusion matrices obtained by combining the feature sets between the models (train- ing/test rate: 0.8/0.2—classifier: SVM): **a** DeiT3 and Swin (I), **b** DeiT3 and ViT (II), **c** Swin and ViT (III), and **d** DeiT3 and Swin and ViT (IV)

**Fig. 17:**
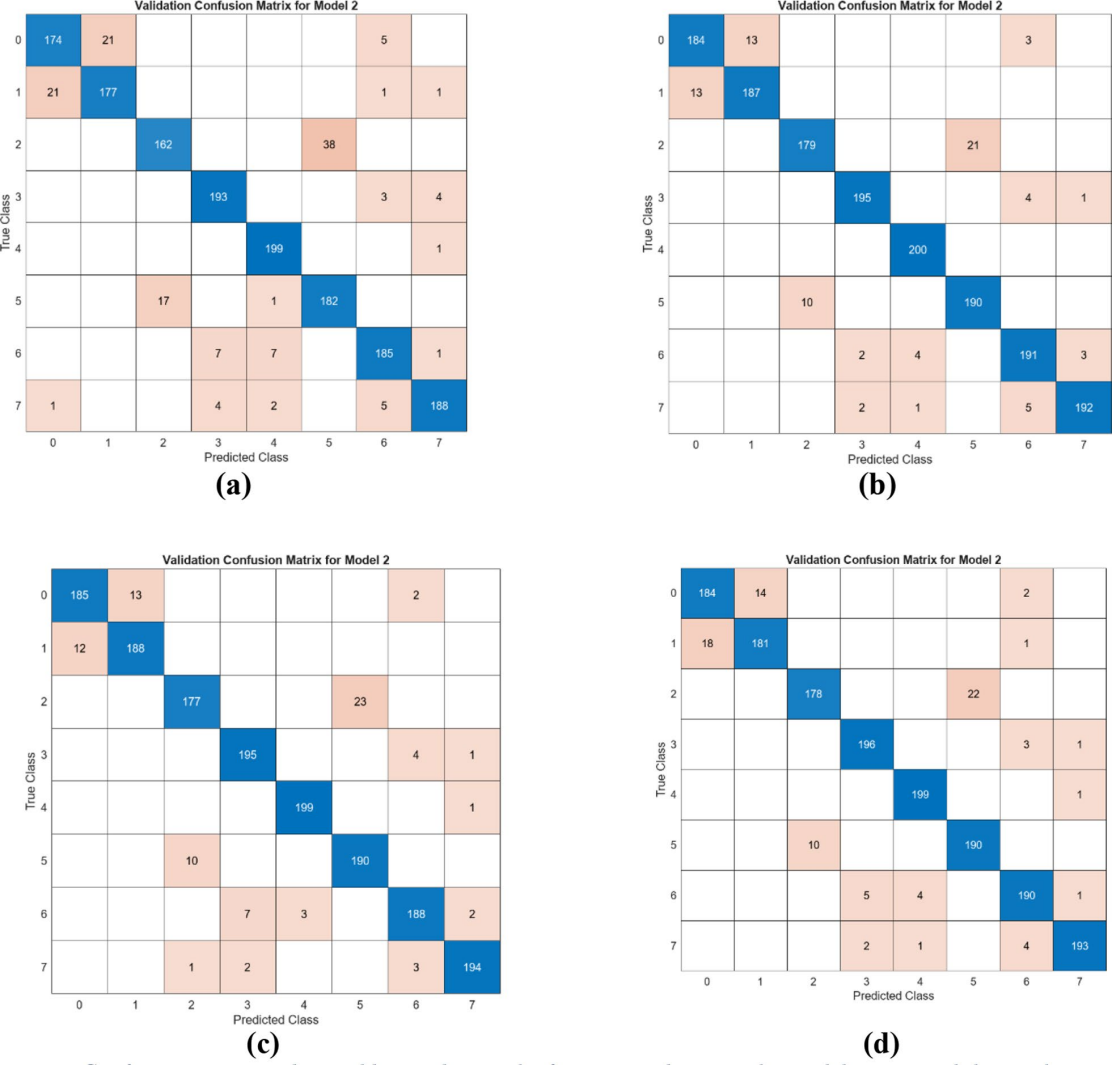
Confusion matrices obtained by combining the feature sets between the models (cross validation: k = 5 and classifier: SVM): **a** DeiT3 and Swin (I), **b** DeiT3 and ViT (II), **c** Swin and ViT (III), and **d** DeiT3 and Swin and ViT (IV)

The performance of the four combined feature sets was assessed using the SVM method. For this evaluation, 20% of the data was allocated for testing, and fivefold cross-validation was employed to ensure robust validation. The confusion matrices generated from the test data are displayed in Fig. 16, while the cross-validation results are shown in Fig. 17. The corresponding metric outcomes for these matrices are summarized in Table 5. In the classification analyses conducted with the training and test data split, the feature set merging between models and the use of SVM achieved the following overall accuracy performances: Model I at 91.4%, Model II at 95.2%, Model III at 95.1%, and Model IV at 94.4%.

**Table 5:** The overall performance accuracy of the mRMR method in classifying the selected features

Combined feature set	Machine learning	Top features	Acc (%) (Test: 0.2)	Acc (%) (Cross val./k = 5)
“II” 1536 features	SVM	100	%92.7	%92.6
		300	%95.3	%94.6
		500	%95.2	%95.1
		700	%95.3	%95.1
		1000	%95.2	%95.0

As shown in Fig. 17, in order to confirm the accuracy of the performance obtained in the study, the dataset was processed using the cross-validation technique (k = 5) and classified with the SVM method. As a result of these operations, Model I achieved an overall accuracy of 91.2%, Model II reached 94.9%, Model III achieved 94.8%, and finally, Model IV reached 94.4%. The cross-validation technique validates the performance of the proposed approach.

**Fig. 18:**
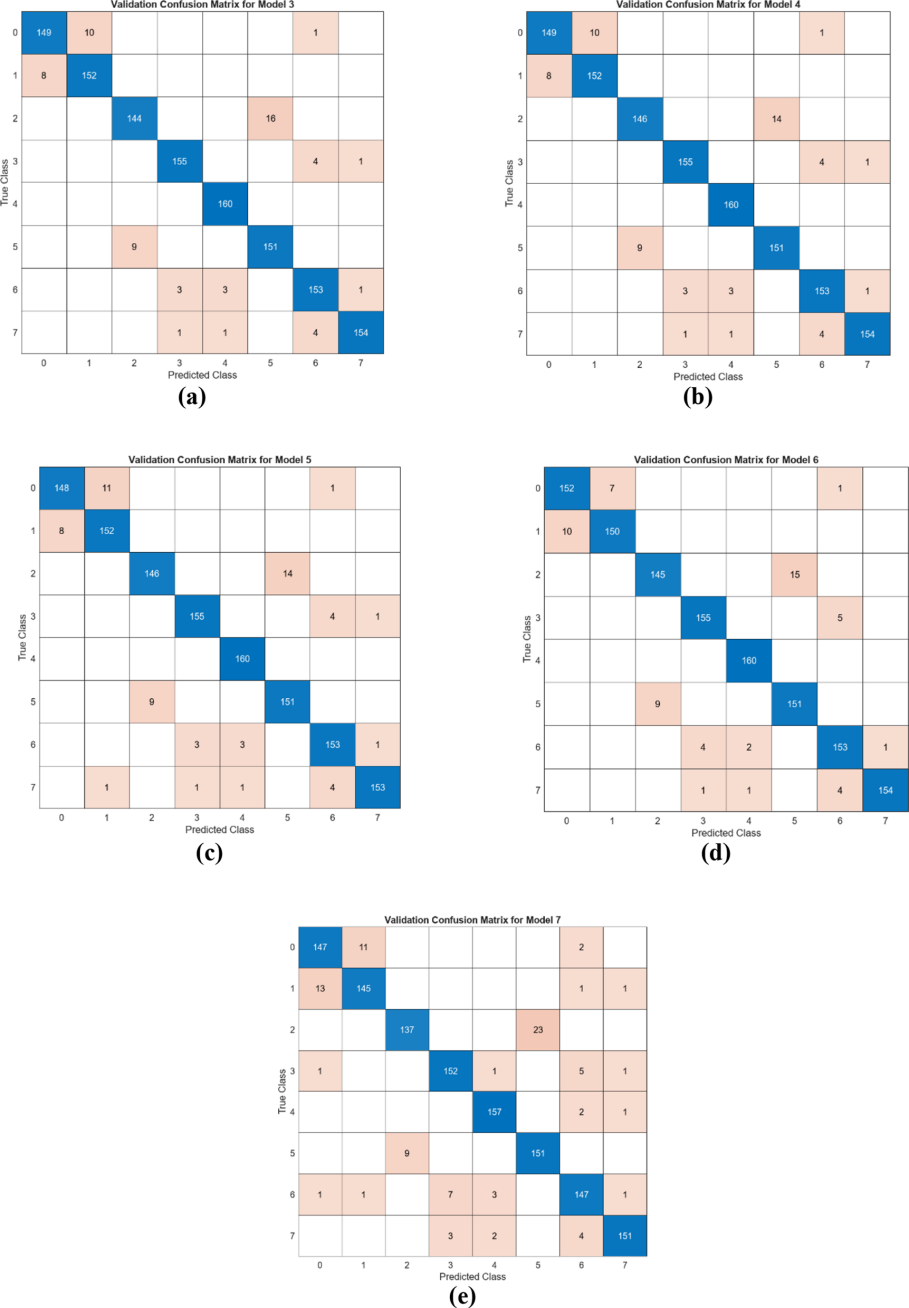
Confusion matrices obtained by SVM classification of the best features selected by mRMR feature selection method (train rate: 0.8, test rate: 0.2): **a** the top 1000 features **b** the top 700 features **c** the top 500 features **d** the top 300 features, **e** the top 100 features

**Fig. 19:**
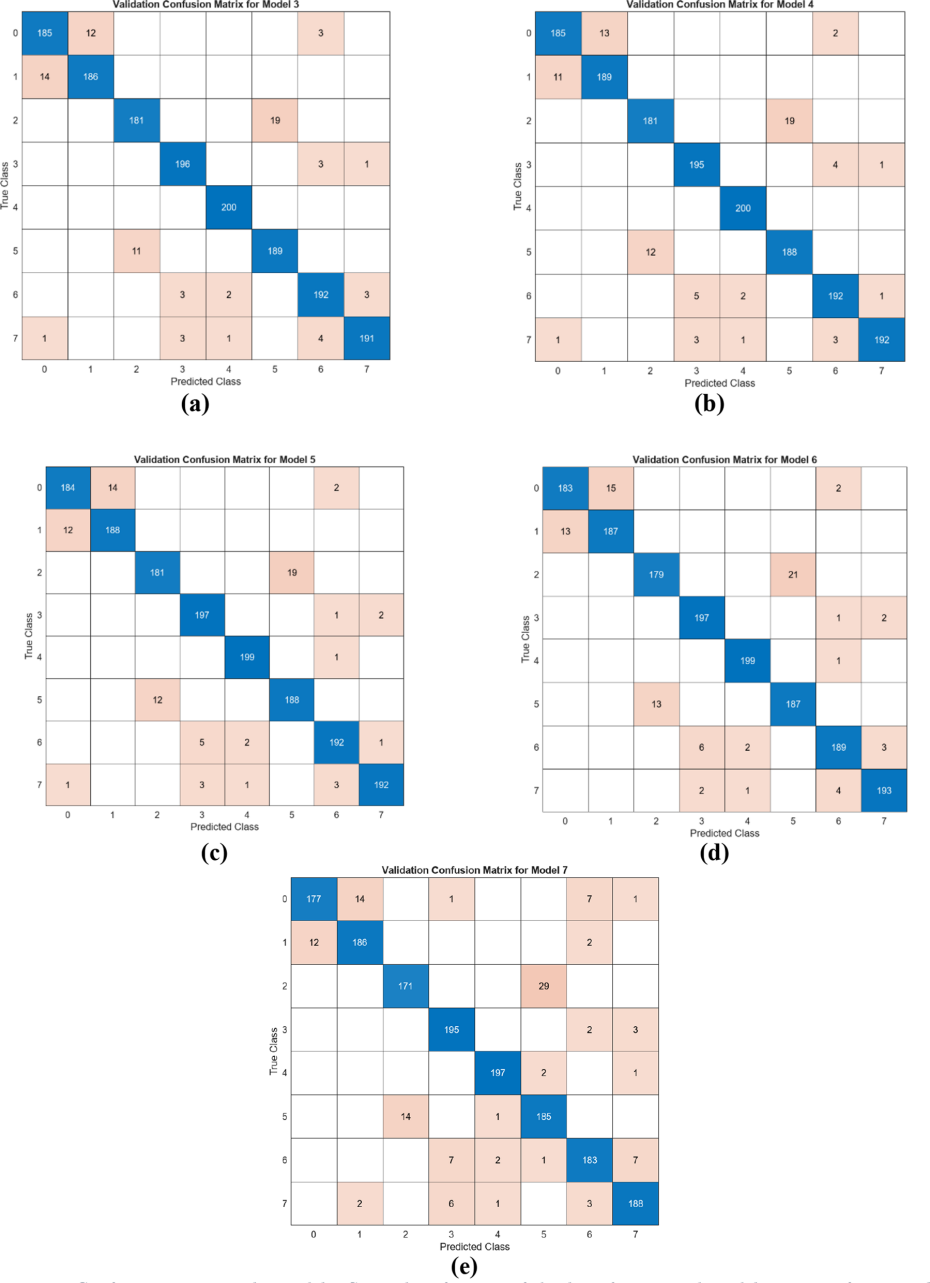
Confusion matrices obtained by SVM classification of the best features selected by mRMR feature selection method (cross validation/k = 5): **a** the top 1000 features **b** the top 700 features **c** the top 500 features **d** the top 300 features, **e** the top 100 features

In the next step of the experimental analysis of the classification study, the objective was to determine whether the feature selection algorithm could achieve better performance with fewer features. When examining the best-performing merged models (I, II, III, and IV), it was observed that Model II (DeiT and ViT) achieved a higher accuracy rate (95.2%) with fewer features (1536). In the subsequent experimental analysis, the feature set that constituted Model II (DeiT + ViT) was analyzed using the mRMR method. The feature selection tool in the MATLAB application was used for the mRMR method. Based on the feature ranking obtained from this application, the best 100, 300, 500, 700, and 1000 feature sets were determined from the Model II feature set. These feature sets were then classified using the SVM method. As in the other steps, the test data was set aside at 20%, and the analysis was carried out using cross-validation (k = 5). In this phase, the results of the analysis conducted with the test data are presented as confusion matrices in Fig. 18. Similarly, the confusion matrices for the analysis conducted with the data separated by cross-validation are shown in Fig. 19. The overall accuracy performance of the ‘II’ feature set determined by the mRMR technique is presented in Table 5.

According to the results obtained in Table 5, it demonstrates that classification performance can be optimized with a feature reduction approach. In the merged dataset consisting of 1536 features, accuracy rates for different numbers of selected features were evaluated using the SVM method. In the analysis with 100 features, an accuracy of 92.7% was achieved on the test set and 92.6% with cross-validation. However, when the number of selected features was increased to 300, the accuracy rate rose to 95.3% on the test set and 94.6% with cross-validation. Similarly high accuracy rates were maintained with 500, 700, and 1000 features, but the best performance of 95.3% accuracy was obtained with the combination of 300 and 700 features. The results show that selecting fewer features can not only increase the model’s efficiency but also maintain or even improve accuracy performance. An increase in the number of features may stabilize accuracy at a certain level while increasing the computational cost of the model. Therefore, selecting 300 or 700 features offers an optimal balance between accuracy and computational cost. By selecting only 300 optimized features instead of using the full feature set, the mRMR algorithm reduces computational cost while maintaining high classification accuracy (95.3%). This allows the model to be more efficient in real-world applications where computational resources are limited.

A comparison of the proposed hybrid model with similar studies using the same dataset is shown in Table 6. In their study, Demirbaş et al. [7] introduced a new architecture called the Spatial-Attention ConvMixer (SAC) model to develop an automatic classification system for gastrointestinal (GI) disorders. The proposed model achieved an accuracy of 93.37%. Ahamed et al. [12] demonstrated a strong performance with the Ensemble Extreme Learning Machine (EELM) classifier, supported by image enhancement techniques, achieving an accuracy of 87.75%. Oğuz et al. [26] proposed a deep learning-based method for the classification of gastrointestinal diseases and colon anatomical markers, achieving an accuracy of 94.125%. Tsai and Lee [27] examined the effectiveness of ensemble learning techniques in convolutional neural network (CNN) models for the classification of gastrointestinal diseases, with their proposed Grad-CAM model achieving an accuracy of 91%.

**Table 6:** Comparison of the proposed hybrid model with existing studies in the literature

Article	Year	Model/method	Result
Demirbaş ve ark. [7]	2024	SAC	Acc: %93.37
Ahamed ve ark [12]	2024	EELM	Acc: %87.75
Oğuz ve ark. [26]	2024	Grad-CAM	Acc: %94.12
Tsai ve Lee [27]	2024	CAM	Acc: %91
Proposed model	2024	ROI and Transformer models and Feature	Acc: %95.2

## Conclusion and future work

### Conclusions

This study developed advanced deep learning-based methods for detecting and classifying gastrointestinal (GI) abnormalities, demonstrating their effectiveness in medical image analysis. The proposed GISegNet segmentation network achieved superior accuracy (93.16%) and segmentation performance, outperforming other models in detecting pathological regions. Additionally, the hybrid classification approach, particularly the combination of DeiT and ViT models, achieved a high accuracy rate of 95.2%, with optimized feature selection via the mRMR algorithm enhancing both efficiency and computational cost. Compared to traditional manual diagnosis, the proposed deep learning system significantly improves diagnostic efficiency by automating segmentation and classification. This reduces the overall workload of gastroenterologists, minimizes diagnostic errors, and ensures a standardized decision-making process. Although the proposed model demonstrates high performance, its generalizability to diverse patient populations remains a limitation due to dataset constraints. Future studies should aim to validate the model on larger, multi-center datasets to ensure robustness across different clinical settings.

### Future work

While this study has demonstrated the effectiveness of deep learning-based methods in detecting and classifying gastrointestinal (GI) abnormalities, several key areas for future research remain. First, testing the proposed models on larger and more diverse datasets will enhance their generalization capability and robustness across different clinical settings. The variability in endoscopic image quality across different institutions presents a challenge for real-world implementation. To address this, future work should explore domain adaptation techniques and collect diverse datasets to improve model adaptability.

For seamless clinical integration, incorporating real-time processing capabilities into endoscopic imaging systems could enable immediate diagnostic insights, reducing the time required for specialist review. Optimizing inference speed and ensuring efficient hardware compatibility will be essential for real-time deployment. Additionally, exploring advanced architectures such as self-supervised and multimodal learning approaches could further improve diagnostic accuracy and efficiency. Finally, close collaboration with medical experts to refine and validate the models in clinical environments will be crucial for ensuring their applicability, reliability, and acceptance in real-world healthcare settings.

## Data Availability

In the conducted study, the training and performance evaluation of the developed models were conducted using publicly available dataset. The dataset used in the study, named “Kvasir” can be found at the following address: https://www.kaggle.com/datasets/abdallahwagih/kvasir-dataset-for-classification-and-segmentation
